# Exploring and Overcoming Challenges for Efficient Audiological Testing in Children Under 5 Years of Age—Screening with Otoacoustic Emissions

**DOI:** 10.3390/audiolres16030074

**Published:** 2026-05-15

**Authors:** Nienke Streefkerk, Franciscus A. Diepstraten, Evangeline A. Huis in ’t Veld, Antoinette am Zehnhoff-Dinnesen, Martine van Grotel, Katrin Neumann, Annelot J. M. Meijer, Frédéric Amant, Ross Parfitt, L’udmila Verešpejová, Penelope R. Brock, Lisa L. Hunter, Hiske W. Helleman, Beth Brooks, Kaukab M. Rajput, Kristin Knight, Marry M. van den Heuvel-Eibrink, Alexander E. Hoetink

**Affiliations:** 1 Princess Máxima Center for Pediatric Oncology, 3584 CS Utrecht, The Netherlands; 2 Department of Phoniatrics and Pediatric Audiology, University Hospital Muenster, University of Muenster, 48149 Muenster, Germany; 3 Gynecological Oncology, Netherlands Cancer Institute, 1066 CX Amsterdam, The Netherlands; 4 Unit of Gynecological Oncology, Department of Oncology, KU Leuven, 3000 Leuven, Belgium; 5 Division of Gynecological Oncology, Department of Obstetrics and Gynecology, UZ Leuven, 3000 Leuven, Belgium; 6 Department of Otorhinolaryngology, 3rd Faculty of Medicine, Charles University Prague, University Hospital Kralovske Vinohrady, 100 34 Prague, Czech Republic; 7 Department of Pediatric Oncology, Great Ormond Street Hospital for Children National Health Service Trust, London WC1N 3WY, UK; 8 Research in Patient Services, Cincinnati Children’s Hospital Medical Center, University of Cincinnati, Cincinnati, OH 45221, USA; 9 Department of Otorhinolaryngology and Head & Neck Surgery, University Medical Center Utrecht, 3584 CX Utrecht, The Netherlands; 10 Department of Audiology, British Columbia’s Children’s Hospital, Vancouver, BC V6H 3N1, Canada; 11 School of Audiology and Speech Science, University of British Columbia, Vancouver, BC V6T 1Z4, Canada; 12 Department of Audiovestibular Medicine and Cochlear Implant, Great Ormond Street Hospital for Children National Health Service Trust, London WC1N 3WY, UK; 13 Department of Pediatric Audiology, Oregon Health and Science University, Portland, OR 97239, USA; 14 Division of Child Health, Wilhelmina Children’s Hospital, University Medical Center Utrecht, 3584 CX Utrecht, The Netherlands; 15 UMC Utrecht Brain Center, Utrecht University, 3584 CX Utrecht, The Netherlands

**Keywords:** ototoxicity, cisplatin exposure, young children, audiology, otoacoustic emissions, pediatric

## Abstract

Background/Objectives: Cisplatin-exposed pediatric cancer patients are at increased risk of ototoxicity, particularly those under 5 years of age. In this group, audiological monitoring is challenging, as interpretation of otoacoustic emissions (OAEs) and tympanometry, used to supplement behavioral audiometry, is subject to interindividual variability. This study aimed to identify key challenges and establish an international consensus on optimal testing procedures and interpretation criteria for assessing early cochlear damage. Methods: Audiological data from 11 children (<5 years) exposed to cisplatin in utero were evaluated. An international panel of 10 pediatric oncology audiology experts reviewed tympanometry, transient evoked OAEs (TEOAEs), and distortion product OAEs (DPOAEs). Areas of disagreement were analyzed, and consensus was sought regarding testing conditions, interpretation, and clinical application. Results: Agreement on cochlear status was good in 10/22 ears, moderate in 3/22 ears, and poor in 9/22 ears, highlighting substantial variability in interpretation. Consensus was achieved on minimal technical and interpretative requirements for OAE testing in this population. The panel proposes a classification framework integrating OAEs and tympanometry to guide clinical follow-up. Importantly, normal OAE results were not considered sufficient to exclude ototoxic damage or mild hearing loss. Conclusions: OAEs, particularly DPOAEs, are valuable as a screening tool in ototoxicity monitoring programs for young children, provided that testing conditions and interpretation are standardized. However, OAEs alone are insufficient for definitive assessment. Longitudinal monitoring and confirmatory testing with behavioral audiometry or ABR/ASSR remain essential. Further validation of the proposed classification system is warranted.

## 1. Introduction

Childhood cancer patients, particularly those treated with platinum derivatives, are at risk of ototoxicity due to the accumulation of platinum in the cochlea [1,2]. This accumulation disrupts DNA replication and transcription and increases reactive oxygen species (ROS), resulting in damage to cochlear outer hair cells and leading to irreversible hearing loss [3,4,5,6,7]. Such hearing loss, especially in young children under the age of 5 years [8], may negatively affect speech and language development, thereby impairing social, emotional, and academic growth and reducing quality of life [9,10]. Early audiological monitoring is therefore crucial for identifying cisplatin-induced hearing loss (CIHL), as timely detection and intervention may help mitigate long-term developmental consequences. Regular hearing assessments are recommended before and after ototoxic chemotherapy and, when feasible, between treatment cycles.

Recently, a consensus on the minimal required audiological testing for children with cancer was published, aiming to standardize the type of testing according to age group [11]. In older children, the gold standard for assessing ototoxic hearing loss consists of a detailed case history and behavioral audiometry, such as Conditioned Play Audiometry (CPA) or Pure Tone Audiometry (PTA). For early detection of ototoxic damage, extended high-frequency audiometry (10–16 kHz) is required, as initial cochlear damage typically occurs in the high-frequency range [12]. There is, however, evidence suggesting that the reliability of high-frequency audiometry in children aged 7 years and younger may be reduced due to a high false-positive rate (24.6%) in younger children [13]. Consequently, reliable testing is particularly challenging in very young children, especially under the age of 3 years, for whom behavioral audiometry is often not feasible.

Hence, in infants and very young children, a combination of behavioral and objective audiological tests is applied according to the “cross-check” principle to ensure reliable assessment [14]. Behavioral tests such as Visual Reinforcement Audiometry (VRA) or CPA may be used but require active engagement of the child. When behavioral audiometry is not yet feasible, the identification and monitoring of ototoxicity become challenging. In such cases, objective measures, including Auditory Brainstem Response (ABR) and Auditory Steady State Response (ASSR), provide valuable information, particularly when frequency regions above 4 kHz are assessed. These tests can be performed during natural sleep or under sedation or anesthesia. Otoacoustic emissions (OAEs) may provide additional objective information on outer hair cell function.

Transient Evoked (TE)OAEs assess cochlear responses to brief click stimuli, with the stimulus and response separated in time, whereas Distortion Product (DP)OAEs detect acoustic signals generated by the cochlea in response to two pure tones, with separation of stimulus and response in the frequency domain. A limitation of OAEs is that they do not allow direct quantification of hearing thresholds. However, DPOAEs offer the advantage of assessing cochlear function at frequencies above 4 kHz, and even beyond 8 kHz, which is particularly relevant for ototoxicity monitoring [15,16,17].

Several international classification systems have been developed to grade ototoxicity in children, most notably the Boston Ototoxicity Scale of the International Society of Paediatric Oncology (SIOP) and the Muenster classification [18,19]. Both are threshold-based systems, designed to enable consistent grading of hearing loss across clinical and research settings and to guide treatment decisions and rehabilitation. Within these systems, ototoxicity is generally considered clinically relevant from grade ≥ 2 in the SIOP Boston classification and from grade ≥ 2b in the Muenster classification.

However, as OAEs do not provide quantifiable hearing thresholds, they cannot be directly incorporated into these grading systems. Consequently, because decisions regarding continuation or modification of cancer treatment are typically based on ototoxicity grades derived from such classifications, OAE results alone are insufficient for clinical decision-making within these frameworks.

Moreover, adequate transmission of sound through the outer and middle ear to the cochlea and back is a prerequisite for reliable interpretation of OAE results [20]. Standard testing conditions may vary across institutes, and interindividual variability in OAE interpretation may therefore arise. To identify and characterize these challenges, we conducted an interpretative exercise in which an international panel of audiological experts in pediatric oncology individually evaluated TEOAEs, DPOAEs, and tympanograms from 11 very young children who were exposed to cisplatin in utero and subsequently audiologically monitored. This exercise aimed to assess concordance and discordance in interpretation, to identify limitations of these audiological tests, and to establish a common framework for minimal testing requirements and consensus-based interpretation of ototoxic damage in this vulnerable age group. Importantly, the objective was not to evaluate the prevalence or severity of hearing loss in this cohort, but to explore expert agreement in audiological interpretation.

## 2. Materials and Methods

For the current study, we gathered a panel of international pediatric oncology audiological experts (from London, UK; Muenster, Germany; Utrecht, the Netherlands; Cincinnati, USA; Portland, USA; and Vancouver, Canada), most of whom contributed to the previous audiological monitoring consensus [11]. They were asked to participate in the exercise to evaluate OAEs and tympanograms of 11 children, and to discuss the results, seeking international consensus on test conditions and interpretation of test results. The primary aim of this study was to assess agreement among experts in the interpretation of audiological findings rather than to evaluate clinical outcomes in the included children.

We gathered a group of 11 children younger than 5 years of age (range: 19–41 months), who had been exposed to cisplatin in utero (due to maternal cancer during pregnancy). These children underwent routine audiological testing as part of their surveillance in the national centralized Cancer in Pregnancy (CIP) offspring clinic in the Princess Máxima Center, as part of the International Cancer in Pregnancy Registry Study (INCIP) (ClinicalTrials.gov; NCT00330447). Written informed consent from their parents was obtained for data provision.

All children initially underwent otoscopy, tympanometry, and TEOAE (up to 4 kHz) as well as DPOAE (up to 10 kHz) recordings. The audiological tests were conducted in a sound-treated booth by trained audiology assistants of the UMC Utrecht audiology department in collaboration with the Princess Máxima Center. Single-frequency tympanometry was used at 226 Hz. OAEs were recorded using a Otodynamics ILOv6 system with an Otodynamics UGD TE+DP OAE probe (Otodynamics Ltd., Hatfield, UK). DPOAEs were elicited using primary tones f1 and f2 at levels of L1/L2 = 65/55 dB SPL and an f2/f1 ratio of 1.22. Responses were recorded at the 2f1–f2 distortion product frequency, with test frequencies presented from high to low. For each frequency, responses were averaged over a maximum of 260 presentations (TEOAE) or a maximum of three minutes (DPOAE), or until a robust OAE response was obtained with the signal-to-noise ratio exceeding 6dB in three adjacent frequencies. Artifact rejection was applied automatically, excluding records with excessive noise with a noise rejection level of 6 dB. All acquisition parameters were kept constant across measurements.

The audiological test results were reviewed by two independent experts (AH and FAD) between 1 December 2021 and 20 December 2021, prior to panel evaluation, to characterize the range of audiological test patterns and interpretation challenges represented in the cohort and relevant to the consensus exercise. No cases were excluded based on this review. All pseudonymized test documentation (see File S1), including evaluation sheets (see File S2), was distributed to the members of the audiological panel in January 2022 for independent assessment. For each ear, the panelists were asked to score the suspicion of hearing loss as one of three predefined categories: “yes”, “no”, or “unclear”. These ratings were returned to the core research team and collected centrally at the Princess Máxima Center.

An introductory meeting was held in 2022, during which the study aims, design, and timeline were presented. After completion of the independent assessments, a series of eight online consensus meetings (Zoom; Zoom Video Communications, San Jose, CA, USA) took place. During these meetings, all 11 cases were discussed systematically. For each case, the following aspects were discussed: (1) independent interpretation of audiological status based on OAE and tympanometry findings; (2) identification of concordant and discordant ratings among panel members; (3) discussion of ambiguities and gaps in the available audiological data; (4) challenges encountered in audiological interpretation; and (5) suggestions to improve data quality, interpretation, and reporting.

Agreement on the presence or absence of suspected hearing loss per ear was quantified based on the proportion of panel members assigning the same category. For descriptive purposes, agreement levels were pragmatically categorized as good (≥75% agreement), moderate (62.5% agreement), and poor ≤50% agreement). Cases demonstrating moderate or poor agreement were subsequently discussed in detail during the consensus meetings until agreement was reached.

Based on group consensus during these discussions, statements were formulated regarding minimal requirements for high-quality audiological testing, principles for the interpretation of OAE and tympanometry results, and recommended follow-up strategies. These statements informed the development of a proposed classification framework to support the interpretation of audiological assessments using OAEs and tympanometry in young pediatric cancer patients.

Inter-rater agreement for the classification of hearing loss was assessed using Gwet’s AC1 for three categorical outcomes (“yes”, “no”, and “unclear”). Ratings for the left and right ears were analyzed both separately and combined, treating each ear as a distinct observation. Because each patient contributed two ears, confidence intervals were estimated using a patient-level cluster bootstrap. Interpretation of agreement coefficients followed conventional benchmarks for chance-corrected agreement measures [21].

## 3. Results

Table 1 shows the panel’s interpretation of the individual cases, based on the assessment of tympanometry and OAE results for suspicion of hearing loss. Overall, agreement was classified as good in 10 of 22 ears, moderate in 3 of 22 ears, and poor in 9 of 22 ears. For right ears, agreement was good in 7 of 11 ears, moderate in 1 of 11 ears, and poor in 3 of 11 ears. For left ears, agreement was good in 3 of 11 ears, moderate in 2 of 11 ears, and poor in 6 of 11 ears.

Inter-rater agreement, quantified using Gwet’s AC1, was 0.26 for the right ear and 0.20 for the left ear. When both ears were pooled, agreement at the ear level was 0.23 (Gwet’s AC1; patient-clustered bootstrap 95% CI 0.09–0.41).

In nine out of 11 cases, the audiological status could be interpreted by the majority of panel members, whereas in two cases this was not possible. The main challenges identified during panel evaluation were incomplete or technically inadequate DPOAE measurements, excessive noise interference during OAE testing, and the presence of middle ear pathology.

In six ears, high-frequency sensorineural hearing loss could not be ruled out because OAEs were present up to 6–8 kHz but absent at 10 kHz despite normal tympanometry.

Panel members were not instructed to apply a predefined tympanometric classification system. Analysis of the panel discussions revealed that different classification approaches were used. Tympanograms were subsequently categorized according to both the ASHA criteria and the Jerger classification, as summarized in Table 2 [22,23]. In three ears, the tympanometric curve was not visible, unavailable, or not interpretable. Using the ASHA grading system, nine ears were classified as normal, four ears were classified as questionable, and six as abnormal [23]. According to the Jerger classification, seven ears were classified as Type A, five as Type As, three as Type B, and four as Type C [22].

Table 3 summarizes the results of follow-up behavioral audiometry using CPA and VRA (see File S3). Follow-up testing excluded hearing loss in three initially uncertain cases and confirmed hearing loss in three children with persistent abnormal findings. In two cases, hearing loss was considered unlikely because findings suggested a conductive component, whereas in one case, additional high-frequency audiometry was considered necessary to evaluate possible high-frequency hearing loss. Two children were lost to follow-up.

## 4. Discussion

Although age-specific recommendations for audiological monitoring in pediatric cancer patients are increasingly available, interpretation of audiological test results remains challenging, particularly in children younger than 5 years. The present study confirms this difficulty, demonstrating that moderate to good agreement on cochlear status based on OAE monitoring was achieved in only 13 of 22 ears (59%). In two of 11 cases (18%), interpretation was precluded by missing or technically invalid OAE data, while in one case (9%), cochlear status could not be assessed due to the presence of conductive hearing loss. These findings are clinically relevant, as undetected ototoxicity may result in long-term sequelae that negatively impact quality of life.

In addition, suboptimal measurement conditions, particularly excessive noise during OAE testing, raised concerns in two of 11 cases (18%), further complicating interpretation. Collectively, these findings illustrate that variability in data quality and the absence of clear interpretative guidance substantially limit the reliability of OAE-based monitoring in this age group.

The results of this consensus exercise indicate that structured interpretation guidance could facilitate more uniform reporting of OAE outcomes and, in turn, improve decision-making regarding appropriate follow-up testing. Such guidance may support earlier and more reliable grading of ototoxicity. Ideally, automated interpretation tools integrating OAE and tympanometry results would assist audiologists and other care providers in consistently identifying children who require further audiological assessment.

While general guidelines for audiological monitoring in pediatric oncology exist, specific recommendations addressing the practical challenges of OAE- and tympanometry-based screening for ototoxicity in platinum-exposed young children are currently lacking. Using real-life clinical cases, the present study brings together the collective experience of an international expert panel in pediatric oncology audiology to examine these challenges and to inform the development of targeted interpretative recommendations.

### 4.1. Interpretation of Audiological Findings

OAEs reflect outer hair cell function and cannot be used to quantify hearing thresholds. Nevertheless, OAEs provide valuable information on cochlear status, particularly in young children in whom behavioral audiometry is not yet feasible. Based on panel consensus, normal TEOAEs up to 4 kHz and DPOAEs up to at least 8 kHz (preferably extending to 10 kHz) were generally interpreted as reassuring for the absence of moderate-to-severe hearing loss within that frequency range. However, the presence of OAEs does not exclude mild hearing loss.

To support more uniform interpretation of OAE and tympanometry results, panel discussions focused on a dichotomous distinction between “no concerns about cochlear status” and “concerns about cochlear status”. Within this framework, “no concerns about cochlear status” corresponded to normal hearing or at most mild hearing loss, whereas “concerns about cochlear status” corresponded to moderate to severe hearing loss. Audiological findings were subsequently translated into a proposed classification framework to support clinical decision-making (Table 4). For this classification, OAEs were considered normal when the signal-to-noise ratio was ≥6 dB in at least three adjacent frequency bands. Based on this proposed classification, the panel considered short-term follow-up with objective or behavioral testing unnecessary when findings raised no concern about cochlear status, whereas findings raising concerns about cochlear status were considered to warrant additional short-term assessment. Independent of this proposed classification, long-term follow-up was considered important in all patients at risk of ototoxicity, also in those with normal OAEs.

Adequate sound transmission through the outer and middle ear is a prerequisite for reliable interpretation of OAE results. The relationship between tympanometric findings and OAE outcomes, therefore, requires careful consideration. Several classification systems exist to define a “normal” tympanogram, including those proposed by ASHA and Jerger [22,23]. However, these systems were primarily developed for screening for otitis media with effusion, rather than for serial monitoring of cochlear function. During serial monitoring for high-frequency hearing loss using DPOAEs, even subtle negative middle ear pressure shifts may influence DPOAE outcomes [24,25].

Based on panel consensus, caution is therefore advised when interpreting changes or decreases in DPOAE amplitude when peak compliance occurs at middle ear pressures below −100 daPa. This threshold aligns with the definition of a Type C tympanogram in the original Jerger classification and was considered more appropriate in the context of ototoxicity monitoring [22].

Based on the technical and interpretative challenges identified during this consensus exercise, the panel formulated several practical considerations regarding test conditions and follow-up assessment.

### 4.2. Practical Considerations for Test Acquisition

High-quality audiological data are a prerequisite for reliable interpretation of OAE and tympanometry results. The expert panel consistently identified technical requirements, optimal testing conditions, and appropriate interaction with young children as essential factors influencing data quality and interpretability. For pediatric oncologists and other caregivers who are less familiar with audiological equipment, it is important to recognize the impact of testing conditions on the reliability of audiological testing.

The panel considered regular maintenance and calibration of audiological equipment important for reliable interpretation, with calibration performed in accordance with manufacturer specifications and at least annually, but more frequently when indicated. Whenever feasible, audiological testing should be conducted in a sound-treated room to minimize the impact of ambient noise on OAE recordings.

In addition, panel members emphasized the importance of adequate training of personnel performing these measurements. This includes competence in achieving and verifying appropriate probe fit, minimizing child- and environment-related noise, and recognizing technical artifacts or equipment malfunction. In cases of technical difficulties, unexpected findings, or questionable measurement quality during tympanometry or OAE testing, repeat measurements were considered advisable to improve data reliability.

### 4.3. Practical Considerations for Follow-Up Testing

The panel agreed that audiological assessment in young children with cancer is best performed in a setting with an experienced technician who is familiar with the specific challenges posed by serious illness and limited cooperation. In clinically fragile patients, prioritization of a minimal set of essential audiological tests was considered preferable, with additional assessments performed when clinically indicated.

For reliable interpretation, the combination of tympanometry and DPOAEs was regarded as important, with DPOAEs measured at least up to 8 kHz and preferably extending to higher frequencies (up to 16 kHz) [26]. When tympanometry suggested a possible conductive component, otoscopy was considered useful to evaluate the ear canal and tympanic membrane and to screen for cerumen impaction or otitis media with effusion. In cases of persistent uncertainty, ear microscopy was considered appropriate. Depending on the clinical context, the panel discussed whether postponement of additional assessment for 6–8 weeks was acceptable or whether immediate behavioral audiometry was preferable.

When OAEs were repeatedly abnormal in the presence of a normal tympanogram, raising suspicion of sensorineural hearing loss, follow-up diagnostics were considered appropriate. These included frequency-specific ABR or ASSR, feasible at all ages and ideally covering frequencies above 4 kHz, and/or behavioral audiometry, when developmentally appropriate (generally from 6 months of age).

Conversely, when tympanometry and TE-/DPOAE findings raised no concerns for hearing loss, the panel generally considered immediate additional behavioral audiological testing unnecessary in the short term. Nevertheless, confirmation of OAE findings by behavioral audiometry during longer-term follow-up was considered advisable.

In cases of inconclusive results, careful evaluation of the test conditions and repetition of measurements were emphasized. As an alternative, particularly when there was clinical suspicion of hearing loss, frequency-specific ABR or ASSR (all ages) and/or behavioral testing (after 6 months of age) were considered appropriate rather than repeated OAE measurements alone [11].

### 4.4. Limitations

This exploratory consensus exercise has several limitations. The children included in this study were exposed to cisplatin in utero, which precluded the availability of baseline audiological assessments prior to exposure. However, the primary aim of this study was not to determine the prevalence of hearing loss, but to evaluate the interpretability and clinical utility of various audiological test modalities for screening and monitoring hearing loss in very young children. The inclusion of a cohort exposed to a known ototoxic risk factor resulted in a population with heterogeneous hearing outcomes, which was considered appropriate for assessing variability in interpretation and consensus formation.

In addition, panel members were not instructed to use a predefined tympanometric classification system during the initial assessment, which may have contributed to inter-rater variability and further highlights the current lack of standardized interpretative approaches in this setting. Tympanograms were categorized according to ASHA and Jerger criteria only retrospectively.

The consensus process involved an international panel of experts and was conducted over an extended time frame to maximize participation.

Future studies should evaluate the proposed classification framework retrospectively in existing cohorts and prospectively in clinical practice to assess its reproducibility, clinical impact, and feasibility. Given the interdisciplinary nature of ototoxicity monitoring, close collaboration between pediatric oncology and audiology communities will be essential to further refine and standardize interpretative approaches.

## 5. Conclusions

This study demonstrates that standardization of audiological test conditions is important and that, despite concordant OAE results in the majority of very young children, interpretation remains challenging in approximately one-third of cases. Through a structured audiological interpretation exercise, consensus was reached on recommended test conditions, combined screening with TEOAEs and DPOAEs in conjunction with otoscopy and tympanometry, and principles for interpreting these findings in individual cisplatin-exposed young children.

This is particularly relevant given the current lack of an internationally standardized approach for interpreting audiological test results and grading hearing damage in this age group. Based on expert consensus, we describe a consensus-based classification framework to support the interpretation of OAE and tympanometry results and to guide decisions regarding appropriate follow-up testing. This framework requires validation in future studies.

Ultimately, longitudinal assessment, in combination with complementary testing modalities such as ABR/ASSR and behavioral audiometry, remains essential to establish definitive hearing status in young children at risk of ototoxicity.

## Figures and Tables

**Table 1:** Audiologists’ hearing assessment based on OAEs + tympanometry.

Case	DPOAEs Present up to		Hearing Loss Right Ear			Hearing Loss Left Ear			Conclusion After Panel Discussion	
	Right Ear	Left Ear	Yes	No	Unclear	Yes	No	Unclear	Right Ear	Left Ear
1	10 kHz	10 kHz	0%	75%	25%	25%	50%	25%	HL unlikely	Conductive HL
2	8 kHz	6 kHz	0%	75%	25%	0%	62.5%	37.5%	Possible HFHL	Possible HFHL
3	8 kHz	8 kHz	25%	37.5%	37.5%	25%	50%	25%	Possible HFHL	Possible HFHL
4	Corrupt	Absent	87.5%	0%	12.5%	87.5%	0%	12.5%	Conductive HL, possible HFHL	Conductive HL, possible HFHL
5	10 kHz	8 kHz	0%	62.5%	37.5%	12.5%	37.5%	50%	HL unlikely	Possible HFHL
6	Absent	Absent	75%	12.5%	12.5%	75%	12.5%	12.5%	Possible HL	Possible HL
7	10 kHz	8 kHz	25%	37.5%	37.5%	0%	50%	50%	HL unlikely	Possible HFHL
8	10 kHz	8 kHz	0%	75%	25%	0%	62.5%	37.5%	HL unlikely	Possible HFHL
9	Not performed	6 kHz	12.5%	12.5%	75%	25%	25%	50%	Unable to assess	Possible HFHL
10	10 kHz	10 kHz	37.5%	25%	37.5%	25%	37.5%	37.5%	HL unlikely	HL unlikely
11	Not performed	Not performed	12.5%	0%	87.5%	0%	0%	100%	Unable to assess	Unable to assess

Audiologists scored OAE + tympanogram on suspicion of hearing loss with ‘yes’, ‘no’, or ‘unclear’. Percentages display the percentage of audiologists choosing this outcome. DPOAE: distortion product otoacoustic emission; HFHL: high frequency hearing loss; HL: hearing loss; OAE: otoacoustic emission.

**Table 2:** Classification of tympanograms following ASHA/Jerger criteria.

Cases			Right Ear				Left Ear			
Case	Sex	Age (Months)	TP (daPa)	TW (daPa)	Jerger Type	ECV (mL)	TP (daPa)	TW (daPa)	Jerger Type	ECV (mL)
1	Female	41	−39.00	87	A	0.50	0.00	NI	B	0.43
2	Female	36	−57.00	111	A	0.42	−90.00	143	A	0.42
3	Female	37	−33.00	145	A	1.05	−27.00	124	A	0.55
4	Male	23	4.00	NI	B	0.54	180.00	NI	B	0.46
5	Female	23	−28.00	163	As	0.43	151.00	153	As ^†^	0.45
6	Female	20	−106.00	187	C	0.42	−150.00	187	As	0.42
7	Female	20	−128.00 ^	121	C	0.62	0.00	119	A	0.59
8	Female	20	−40.00	171	As	0.43	−18.00	143	As	0.46
9	Male	19	−84.00	110	A	0.52	NA	NA	NA	NA
10	Male	19	0.00 ^	99	C	0.62	−205.00 ^&^	99	C	0.60
11	Male	24	NI	NI	B	3.45	NI	NI	B ^†^	4.56

ECV: external canal volume; NA: not available; NI: not interpretable; TP: tympanometric pressure; TW: tympanometric width. ^ no +200 daPa tail to peak; & peak not near atmospheric pressure; † artifacts present. Probe tone frequency was 226 Hz in all cases. Jerger classifications were based on overall tympanometric curve morphology and not solely on tympanometric pressure values. All tympanograms are provided in File S1.

**Table 3:** Follow-up audiological testing.

Case	Age at Follow-Up	Audiological Assessment	Tympanogram (Jerger)		Muenster Classification		SIOP Classification	
			Right	Left	Right	Left	Right	Left
1	NA	NA	NA	NA	NA	NA	NA	NA
2	3 y	PTA	A	A	≤2a	≤1	<2	0
3	6 y 3 m	PTA	A	As	0	0	0	0
4	5 y 11 m	PTA	A	A	2a	1	1	0
5	NA	NA	NA	NA	NA	NA	NA	NA
6	3 y 2 m	VRA	B	B	≤2c	≤2c	≤1	≤1
7	3 y 2 m	VRA	A	A	2a	2b	1	2
8	3 y 3 m	VRA	B	C	≤1	≤1	0	≤1
9	3 y 3 m	VRA	A	A *	1	2a	0	1
10	3 y 3 m	VRA	C *	C	1	≤2a	0	≤1
11	3 y	VRA	NV	C	≤1	≤1	≤1	≤1

m: months; NA: not assessed; NV: curve not visible; PTA: pure tone audiometry; VRA: visually reinforcement audiometry; y: years. *: atypical curve.

**Table 4:** Proposed classification of tympanograms and OAEs per ear.

	Conclusion	Tympanogram	TEOAE/DPOAE (High Frequency) *
**0**	No concerns about cochlear status	Type A	Normal (incl. high frequency)
**1a**	No concerns about cochlear status, but repetition of OAE testing is necessary	Type A	DPOAE normal and TEOAE corrupt
**1b**	Mild conductive hearing loss, but no concerns about cochlear status	Type B/C	Normal (incl. high frequency)
**2a**	Cochlear status unknown, repetition of OAE testing is necessary	Type A/B/C	Both measurements corrupt/missing
**2b**	Conductive hearing loss that prevents assessment of cochlear status, repeat OAE testing after 6–8 weeks or short-term follow-up with ABR/ASSR or behavioral audiometry	Type B	Both measurements abnormal/absent
**3a**	Concerns about cochlear status, repetition of OAE testing necessary, or short-term follow-up with ABR/ASSR or behavioral audiometry	Type A/B/C	At least one measurement abnormal, and one corrupt
**3b**	Concerns about cochlear status, short-term follow-up with ABR/ASSR or behavioral audiometry	Type A	At least one out of two measurements abnormal/absent (or only abnormal/absent in high frequency)

* OAEs are considered normal when the signal-to-noise ratio is ≥6 dB in at least three adjacent frequency bands.

## Data Availability

The original contributions presented in this study are included in the article. Further inquiries can be directed to the corresponding author.

## Supplementary Materials

The following supporting information can be downloaded at: https://www.mdpi.com/article/10.3390/audiolres16030074/s1, File S1: Audiological assessments per patient; File S2: Evaluation sheet sent out to audiological experts; File S3: Follow-up audiological assessments per patient.

## Abbreviations

The following abbreviations are used in this manuscript:

OAE	Otoacoustic emission
DPOAE	Distortion product otoacoustic emission
TEOAE	Transient evoked otoacoustic emission
ABR	Auditory brainstem response
ASSR	Auditory steady state response
CPA	Conditioned play audiometry
VRA	Visual reinforcement audiometry
PTA	Pure tone audiometry
