# Supplementary material for: Exploring and Overcoming Challenges for Efficient Audiological Testing in Children Under 5 Years of Age—Screening with Otoacoustic Emissions
Source: Audiol Res. 2026 May 15;16(3):74. doi: 10.3390/audiolres16030074 (PMC13214472; doi:10.3390/audiolres16030074)
Supplement: Supplementary file 1 [file audiolres-16-00074-s001.zip › File_S1.pdf]

Patient ID: 01

Sex: female

Age: 41 months

Supplement 1. Audiological assessments per patient

DP-OAE Test Report – PID 01

Right ear

Ear canal frequency response

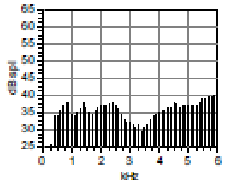

DPOAE response

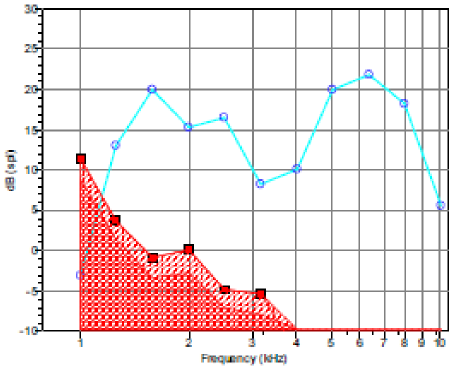

Test Summary
Sum all 1/2 octave = 25,0dBspl
Ave DP 1/2oct (1-6) = 16,0dBspl

Half octave band OAE power

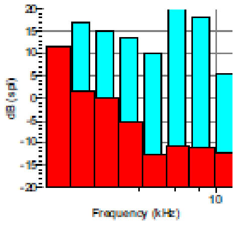

Freq (kHz)	Signal (dBspl)	Noise (dBspl)	SNR (dB)
1.0	-3,1	11,5	-14,6
1.4	17,2	1,7	15,5
2.0	15,3	0,1	15,2
2.8	13,4	-5,1	18,5
4.0	10,2	-12,5	22,7
6.0	21,0	-10,7	31,7
8.0	18,3	-11,1	29,4
10.0	5,6	-12,1	17,7

Test Environment
NLo = 454 NHi = 106 RejLev = 8mPa, 52,0dBspl Test time = 55s
Hardware= USBOAE Probe = Probe 1

Left ear

Ear canal frequency response

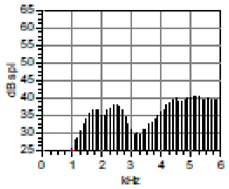

DPOAE response

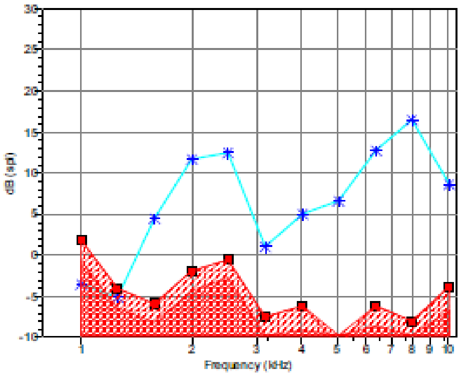

Test Summary
Sum all 1/2 octave = 19,5dBspl
Ave DP 1/2oct (1-6) = 10,5dBspl

Half octave band OAE power

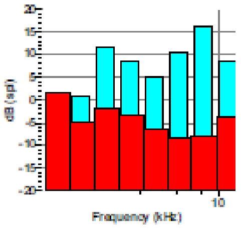

Freq (kHz)	Signal (dBspl)	Noise (dBspl)	SNR (dB)
1.0	-3,4	1,8	-5,2
1.4	1,0	-4,9	5,9
2.0	11,8	-1,9	13,7
2.8	8,6	-3,4	12,0
4.0	5,1	-6,2	11,3
6.0	10,3	-8,2	18,5
8.0	16,5	-8,0	24,5
10.0	8,7	-3,8	12,5

Test Environment
NLo = 567 NHi = 153 RejLev = 8mPa, 52,0dBspl Test time = 70s
Hardware= USBOAE Probe = Probe 1

Right

Freq Hz	L1 dBspl	L2 dBspl	DP dBspl	2SD Noise dBspl	1SD Noise dBspl	SNR dB
1001	58,7	53,9	-3,1	11,5	9,0	-14,6
1257	62,0	54,8	13,1	3,7	1,4	9,4
1587	64,5	54,4	20,0	-0,8	-3,3	20,8
2002	63,8	55,1	15,3	0,1	-2,6	15,2
2515	65,4	52,9	16,6	-4,8	-7,0	21,4
3174	62,7	55,0 *	8,2	-5,4	-7,9	13,6
4004	65,0 *	53,7	10,2	-12,5	-14,9	22,7
5042	63,9	55,5	20,0	-10,8	-13,0	30,8
6348	67,2	56,9	21,9	-10,6	-13,7	32,5
7996	66,8	55,0 *	18,3	-11,1	-13,5	29,4
10083	67,3	55,0 *	5,6	-12,1	-14,7	17,7

Left

Freq Hz	L1 dBspl	L2 dBspl	DP dBspl	2SD Noise dBspl	1SD Noise dBspl	SNR dB
1001	65,0 *	55,0 *	-3,4	1,8	-0,6	-5,2
1257	62,9	53,7	-5,0	-4,0	-6,5	-1,0
1587	65,2	55,0	4,5	-5,9	-8,2	10,4
2002	66,0	53,9	11,8	-1,9	-4,2	13,7
2515	65,7	53,4	12,6	-0,6	-2,7	13,2
3174	63,9	55,0 *	1,1	-7,5	-10,0	8,6
4004	65,0 *	55,2	5,1	-6,2	-9,1	11,3
5042	66,1	55,7	6,7	-11,0	-13,5	17,7
6348	65,7	56,4	12,8	-6,1	-8,5	18,9
7996	67,3	57,6	16,5	-8,0	-9,7	24,5
10083	66,8	55,5	8,7	-3,8	-6,7	12,5

Supplement 1. Audiological assessments per patient

TEOAE Test Report – PID 01

Right

Test type: TE - QuickScreen
Stimulus: 83,7 dB peSPL
Mode: Gen Diag
Tester ID: AUD
Data file: 1ROV4S34.DTA
Notes:

Response waveform

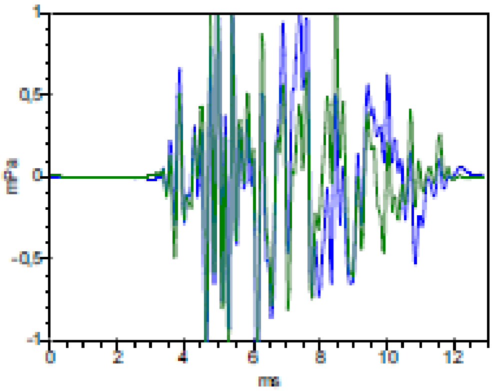

Half octave band OAE power

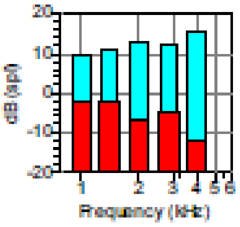

Freq (kHz)	Signal (dB spl)	Noise (dB spl)	SNR (dB)
1.0	9,6	-2,6	12,3
1.4	10,6	-1,9	12,5
2.0	12,5	-6,5	19,0
2.8	12,1	-5,1	17,2
4.0	15,6	-11,8	27,4

Test Summary
Total OAE response = 19,6dBspl   Total Noise = 14,0dBspl

Checkfit stimulus

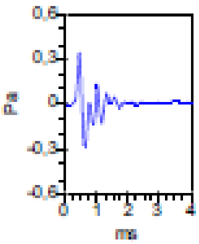

Ear canal response

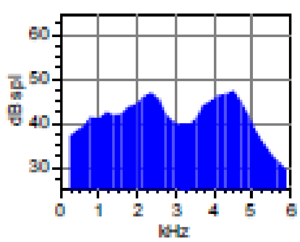

Test Environment

NLo = 160   NH = 96   Test time = 54s
RejLev = 52,0dBspl   Repro =79%   Stim stab = 95%
Hardware:USBOAE   Probe = Probe 1

Left

Test type: TE - QuickScreen
Stimulus: 84,4dB peSPL
Mode: Gen Diag
Tester ID: AUD
Data file: 1ROV4S36.DTA
Notes:

Response waveform

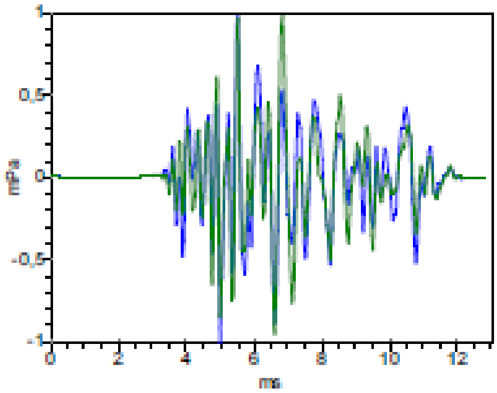

Half octave band OAE power

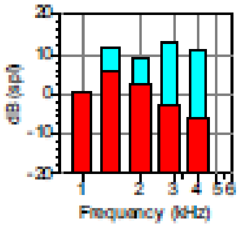

Freq (kHz)	Signal (dB spl)	Noise (dB spl)	SNR (dB)
1.0	-0,7	0,6	-1,2
1.4	11,4	5,5	5,9
2.0	9,0	2,4	6,5
2.8	12,9	-2,9	15,7
4.0	10,7	-6,0	16,7

Test Summary
Total OAE response = 17,2dBspl   Total Noise = 9,3dBspl

Checkfit stimulus

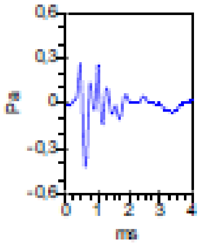

Ear canal response

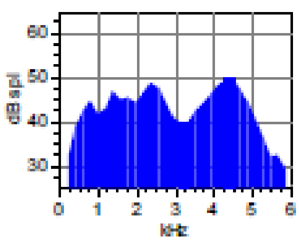

Test Environment

NLo = 260   NH = 108   Test time = 81s
RejLev = 52,0dBspl   Repro =87%   Stim stab = 91%
Hardware= USBOAE   Probe = Probe 1

Supplement 1. Audiological assessments per patient

Tympanometry – PID 01

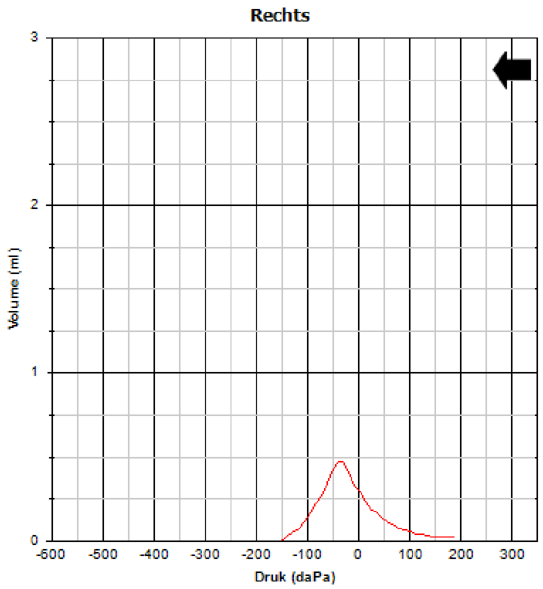

**ECV.**	0,50 ml	**Begin druk**	200,00 daPa
**Compliantie**	0,47 ml	**Eind druk**	-400,00 daPa
**Druk**	-39,00 daPa	**Pomp snelheid**	Maximaal
**Gradiënt**	0,24 ml	**Test toon**	226 Hz

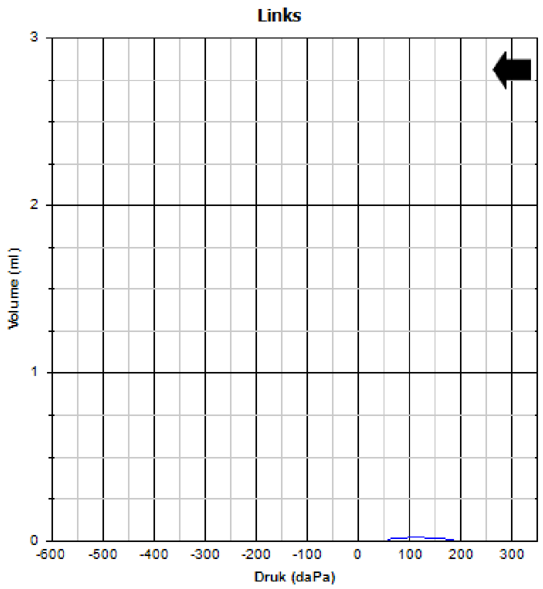

**ECV.**	0,43 ml	**Begin druk**	200,00 daPa
**Compliantie**	32,70 ml	**Eind druk**	-400,00 daPa
**Druk**	0,00 daPa	**Pomp snelheid**	Maximaal
**Gradiënt**	32,70 ml	**Test toon**	226 Hz

Patient ID: 02

Sex: female

Age: 36 months

Supplement 1. Audiological assessments per patient

DP-OAE Test Report – PID 02

Right

Ear canal frequency response

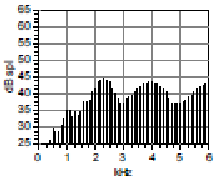

DPOAE response

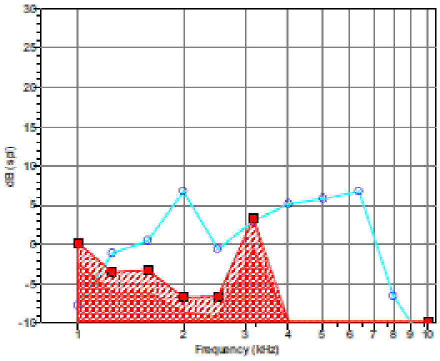

Test Summary
Sum all 1/2 octave = 11,7dBspl
Ave DP 1/2oct (1-6) = 2,7dBspl

Half octave band OAE power

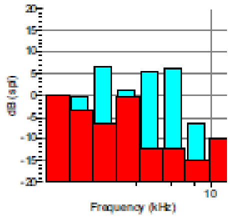

Freq (kHz)	Signal (dBspl)	Noise (dBspl)	SNR (dB)
1.0	-7,7	0,2	-7,9
1.4	-0,2	-3,3	3,1
2.0	6,8	-6,6	13,4
2.8	1,5	-0,2	1,7
4.0	5,2	-12,4	17,6
6.0	6,3	-12,4	18,7
8.0	-6,5	-14,7	8,2
10.0	-13,3	-9,9	-3,4

Test Environment
NLo = 964 NHi = 108 RejLev = 8mPa, 52,0dBspl Test time = 102s
Hardware= USBOAE Probe = Probe 1

Left

Ear canal frequency response

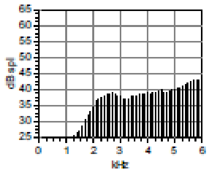

DPOAE response

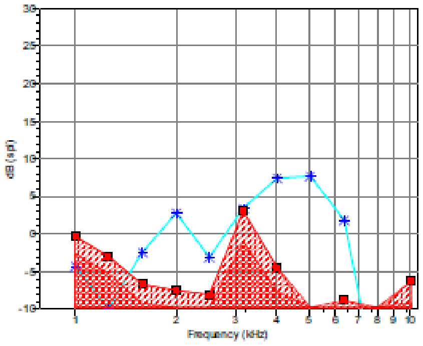

Test Summary
Sum all 1/2 octave = 10,8dBspl
Ave DP 1/2oct (1-6) = 1,8dBspl

Half octave band OAE power

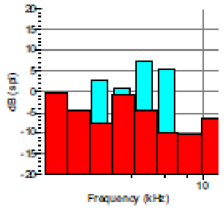

Freq (kHz)	Signal (dBspl)	Noise (dBspl)	SNR (dB)
1.0	-4,3	-0,2	-4,1
1.4	-5,4	-4,6	-0,7
2.0	2,8	-7,4	10,2
2.8	0,8	-0,8	1,6
4.0	7,5	-4,4	11,9
6.0	5,3	-9,9	15,3
8.0	-20,4	-10,1	-10,3
10.0	-15,1	-6,3	-8,8

Test Environment
NLo = 926 NHi = 274 RejLev = 8mPa, 52,0dBspl Test time = 114s
Hardware= USBOAE Probe = Probe 1

Right

Freq Hz	L1 dBspl	L2 dBspl	DP dBspl	2SD Noise dBspl	1SD Noise dBspl	SNR dB
1001	55,9	47,3	-7,7	0,2	-2,3	-7,9
1257	58,9	48,0	-1,0	-3,5	-6,0	2,5
1587	59,8	52,3	0,5	-3,2	-6,0	3,7
2002	62,4	53,8	6,8	-6,6	-8,5	13,4
2515	63,8	52,1	-0,5	-6,5	-9,0	6,0
3174	62,9	55,0 *	3,1	3,4	-0,4	-0,3
4004	63,2	53,4	5,2	-12,4	-15,2	17,6
5042	63,3	55,0 *	5,8	-14,1	-16,6	19,9
6348	62,9	55,6	6,8	-10,9	-13,3	17,7
7996	65,7	55,5	-6,5	-14,7	-16,9	8,2
10083	65,8	57,9	-13,3	-9,9	-12,2	-3,4

Left

Freq Hz	L1 dBspl	L2 dBspl	DP dBspl	2SD Noise dBspl	1SD Noise dBspl	SNR dB
1001	65,0 *	55,0 *	-4,3	-0,2	-2,5	-4,1
1257	65,0 *	55,0 *	-9,9	-3,0	-5,2	-6,9
1587	63,1	55,8	-2,4	-6,6	-9,1	4,2
2002	67,5	55,8	2,8	-7,4	-10,0	10,2
2515	65,8	53,8	-3,1	-8,0	-10,5	4,9
3174	64,9	53,5	3,5	3,1	-0,9	0,4
4004	64,1	53,4	7,5	-4,4	-7,6	11,9
5042	64,1	55,7	7,8	-11,2	-13,8	19,0
6348	66,4	56,1	1,9	-8,8	-11,4	10,7
7996	66,0	56,0	-20,4	-10,1	-12,3	-10,3
10083	67,2	55,0 *	-15,1	-6,3	-9,0	-8,8

TEOAE Test Report – PID 02

Right

Test type: TE - QuickScreen
Stimulus: 90,0 dB peSPL
Mode: Gen Diag
Tester ID: AUD
Data file: 1ROV9F30.DTA
Notes:

Response waveform

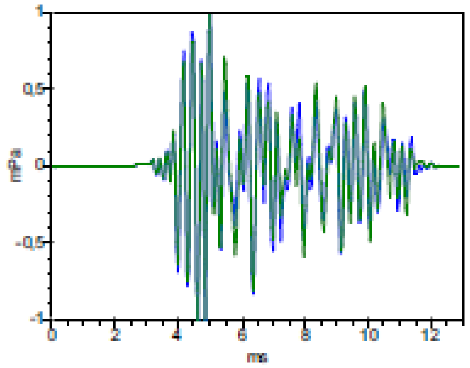

Half octave band OAE power

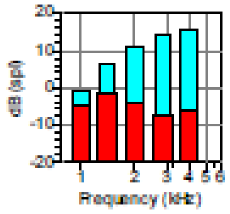

Freq (kHz)	Signal (dB spl)	Noise (dB spl)	SNR (dB)
1.0	-1,2	-4,7	3,6
1.4	6,2	-1,8	8,0
2.0	10,6	-4,2	14,8
2.8	14,1	-7,8	21,8
4.0	15,3	-6,2	21,5

Test Summary

Total OAEresponse = 18,8dBspl Total Noise = 3,6dBspl

Checkfit stimulus

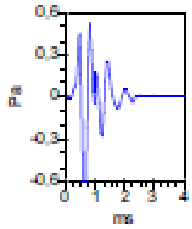

Ear canal response

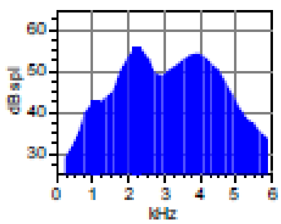

Test Environment

NLo = 260 NH = 20 Test time = 70s
RejLev = 52,0dBspl Repro = 97% Stim stab = 99%
Hardware=USBOAE Probe = Probe 1

left

Testtype: TE - QuickScreen
Stimulus: 83,5dB peSPL
Mode: Gen Diag
Tester ID: AUD
Data file: 1ROV9F32.DTA
Notes:

Response waveform

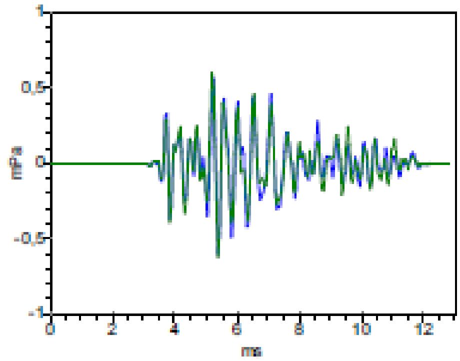

Half octave band OAE power

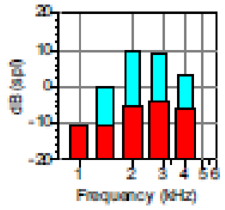

Freq (kHz)	Signal (dBspl)	Noise (dBspl)	SNR (dB)
1.0	-22,9	-10,9	-12,0
1.4	-0,4	-11,0	10,7
2.0	9,3	-5,8	15,1
2.8	8,8	-4,7	13,5
4.0	3,2	-6,2	9,4

Test Summary

Total OAEresponse = 12,8dBspl Total Noise = 1,6dBspl

Checkfit stimulus

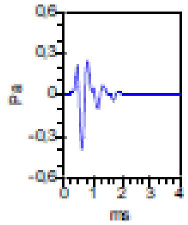

Ear canal response

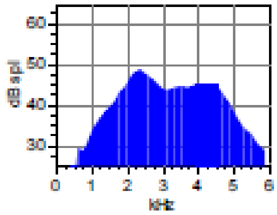

Test Environment

NLo = 260 NH = 5 Test time = 68s
RejLev = 52,0dBspl Repro = 93% Stim stab = 95%
Hardware=USBOAE Probe = Probe 1

Supplement 1. Audiological assessments per patient

Tympanometry – PID 02

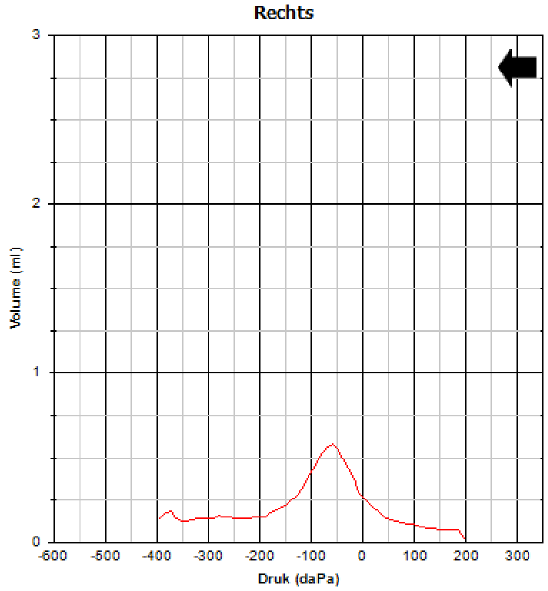

**ECV.**	0,42 ml	**Begin druk**	200,00 daPa
**Compliantie**	0,58 ml	**Eind druk**	-400,00 daPa
**Druk**	-57,00 daPa	**Pomp snelheid**	Maximaal
**Gradiënt**	0,20 ml	**Test toon**	226 Hz

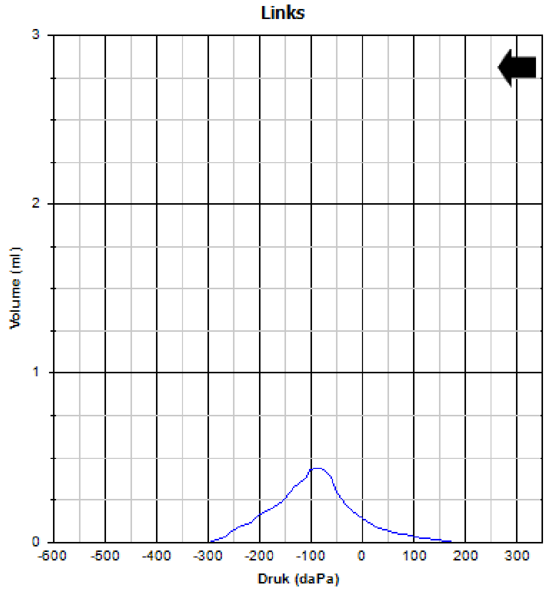

**ECV.**	0,42 ml	**Begin druk**	200,00 daPa
**Compliantie**	0,44 ml	**Eind druk**	-400,00 daPa
**Druk**	-90,00 daPa	**Pomp snelheid**	Maximaal
**Gradiënt**	0,14 ml	**Test toon**	226 Hz

Patient ID: 03

Sex: female

Age: 37 months

Supplement 1. Audiological assessments per patient

DP-OAE Test Report – PID 03

Right ear

Ear canal frequency response

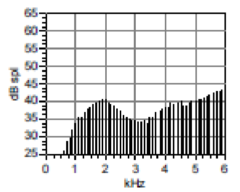

DPOAE response

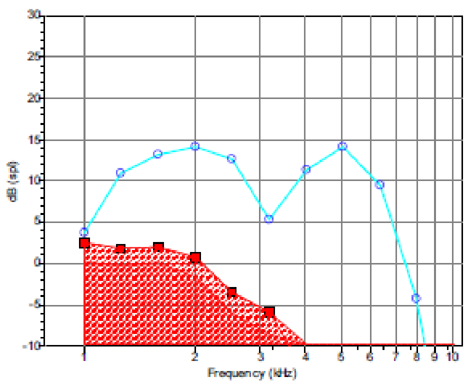

Test Summary
Sum all 1/2 octave = 19,3dBspl
Ave DP 1/2oct (1-6) = 10,2dBspl

Half octave band OAE power

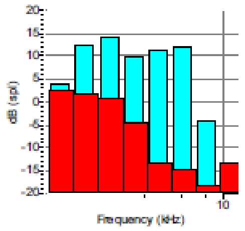

Freq (kHz)	Signal (dBspl)	Noise (dBspl)	SNR (dB)
1.0	3,8	2,5	1,3
1.4	12,2	2,0	10,2
2.0	14,1	0,8	13,3
2.8	9,8	-4,6	14,3
4.0	11,3	-13,5	24,8
6.0	12,1	-14,7	26,8
8.0	-4,2	-18,3	14,1
10.0	-30,0	-13,6	-16,4

Test Environment
NLo = 1009 NHi = 111 RejLev = 8mPa, 52,0dBspl Test time = 106s
Hardware= USBOAE Probe = Probe 1

Left ear

Ear canal frequency response

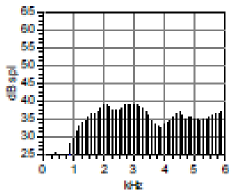

DPOAE response

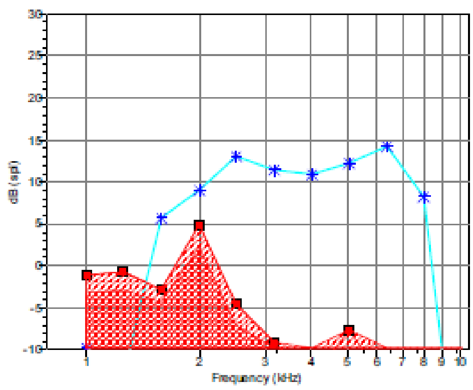

Test Summary
Sum all 1/2 octave = 18,3dBspl
Ave DP 1/2oct (1-6) = 9,2dBspl

Half octave band OAE power

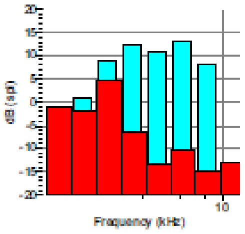

Freq (kHz)	Signal (dBspl)	Noise (dBspl)	SNR (dB)
1.0	-9,7	-1,0	-8,7
1.4	0,6	-1,7	2,3
2.0	9,0	4,9	4,1
2.8	12,3	-6,5	18,8
4.0	10,9	-13,3	24,2
6.0	13,4	-10,3	23,6
8.0	8,3	-14,7	23,0
10.0	-30,0	-13,0	-17,0

Test Environment
NLo = 919 NHi = 153 RejLev = 8mPa, 52,0dBspl Test time = 102s
Hardware= USBOAE Probe = Probe 1

Right

Freq Hz	L1 dBspl	L2 dBspl	DP dBspl	2SD Noise dBspl	1SD Noise dBspl	SNR dB
1001	61,1	55,1	3,8	2,5	0,3	1,3
1257	65,3	55,3	11,0	1,9	-0,2	9,1
1587	66,1	55,3	13,2	2,0	0,1	11,2
2002	65,1	55,4	14,1	0,8	-1,6	13,3
2515	64,6	51,1	12,7	-3,5	-5,9	16,2
3174	65,0 *	55,0 *	5,3	-5,8	-8,5	11,1
4004	65,0 *	53,6	11,3	-13,5	-15,4	24,8
5042	64,0	56,9	14,1	-15,7	-18,1	29,8
6348	64,9	57,4	9,5	-13,8	-16,6	23,3
7996	67,6	57,7	-4,2	-18,3	-20,9	14,1
10083	67,6	55,0 *	-30,0	-13,6	-16,2	-16,4

Left

Freq Hz	L1 dBspl	L2 dBspl	DP dBspl	2SD Noise dBspl	1SD Noise dBspl	SNR dB
1001	65,0 *	55,0 *	-9,7	-1,0	-3,2	-8,7
1257	65,0 *	53,6	-13,2	-0,7	-2,8	-12,5
1587	64,2	54,4	5,7	-2,8	-4,3	8,5
2002	64,5	55,2	9,0	4,9	2,2	4,1
2515	64,8	54,2	13,1	-4,5	-7,1	17,6
3174	64,7	53,1	11,5	-9,1	-11,5	20,6
4004	64,2	55,0 *	10,9	-13,3	-16,0	24,2
5042	65,0 *	55,0 *	12,3	-7,6	-9,8	19,9
6348	65,0 *	56,4	14,3	-14,1	-16,5	28,4
7996	66,0	56,5	8,3	-14,7	-16,9	23,0
10083	65,2	57,4	-30,0	-13,0	-14,4	-17,0

Supplement 1. Audiological assessments per patient

TEOAE Test Report – PID 03

Right

Test type: TE - QuickScreen
Stimulus: 86,8dB peSPL
Mode: Gen Diag
Tester ID: AUD
Data file: 1ROSBL32.DTA
Notes:

Response waveform

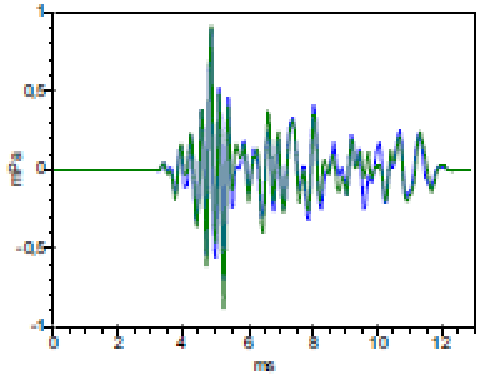

Half octave band OAE power

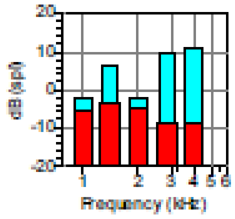

Freq (kHz)	Signal (dB spl)	Noise (dBspl)	SNR (dB)
1.0	-2,1	-5,8	3,7
1.4	6,4	-3,2	9,5
2.0	-2,6	-5,0	2,3
2.8	9,3	-9,0	18,3
4.0	10,6	-8,4	19,0

Test Summary
Total OAEresponse = 14,1dBspl Total Noise = 2,4dBspl

Checkfit stimulus

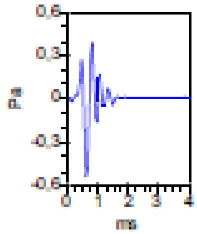

Ear canal response

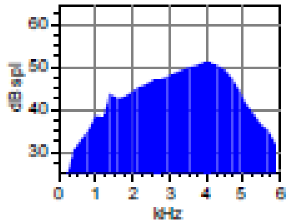

Test Environment
NLo = 260 NH = 5 Test time = 68s
RejLev = 52,0dBspl Repro = 93% Stim stab = 84%
Hardware: USBOAE Probe = Probe 1

Left

Test type: TE - QuickScreen
Stimulus: 85,4dB peSPL
Mode: Gen Diag
Tester ID: AUD
Data file: 1ROSBL34.DTA
Notes: 8460379

Response waveform

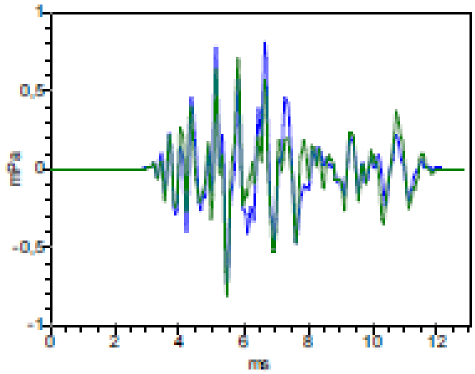

Half octave band OAE power

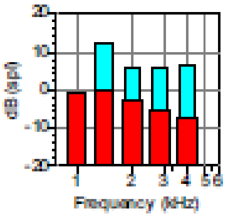

Freq (kHz)	Signal (dBspl)	Noise (dBspl)	SNR (dB)
1.0	-1,8	-1,0	-0,8
1.4	12,0	0,1	11,9
2.0	5,8	-3,0	8,8
2.8	5,3	-5,2	10,5
4.0	6,1	-7,9	14,1

Test Summary
Total OAEresponse = 14,3dBspl Total Noise = 6,6dBspl

Checkfit stimulus

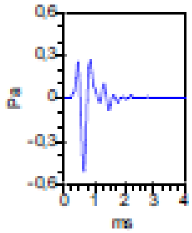

Ear canal response

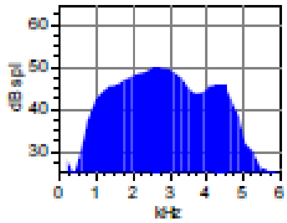

Test Environment
NLo = 229 NH = 66 Test time = 68s
RejLev = 52,0dBspl Repro = 86% Stim stab = 98%
Hardware= USBOAE Probe = Probe 1

Tympanometry – PID 03

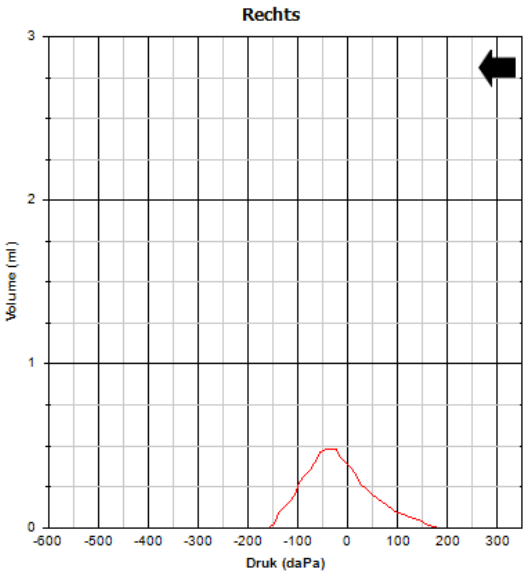

**ECV.**	1,05 ml	**Begin druk**	200,00 daPa
**Compliantie**	0,48 ml	**Eind druk**	-400,00 daPa
**Druk**	-33,00 daPa	**Pomp snelheid**	Maximaal
**Gradiënt**	0,14 ml	**Test toon**	226 Hz

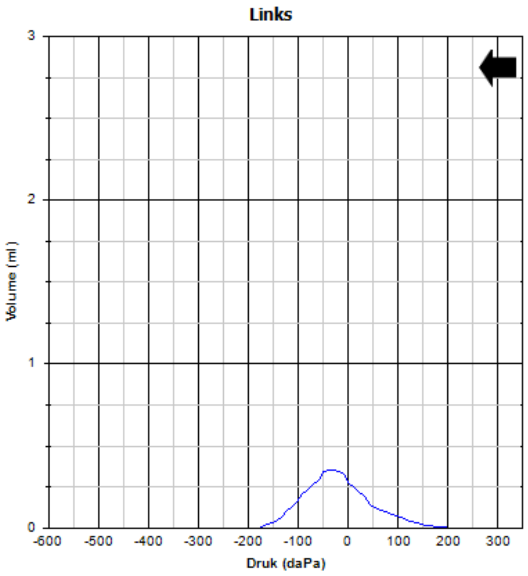

**ECV.**	0,55 ml	**Begin druk**	200,00 daPa
**Compliantie**	0,35 ml	**Eind druk**	-400,00 daPa
**Druk**	-27,00 daPa	**Pomp snelheid**	Maximaal
**Gradiënt**	0,11 ml	**Test toon**	226 Hz

Patient ID: 04

Sex: male

Age: 23 months

Supplement 1. Audiological assessments per patient

DP-OAE Test Report – PID 04

Right ear

Ear canal frequency response

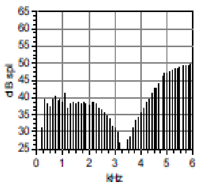

DPOAE response

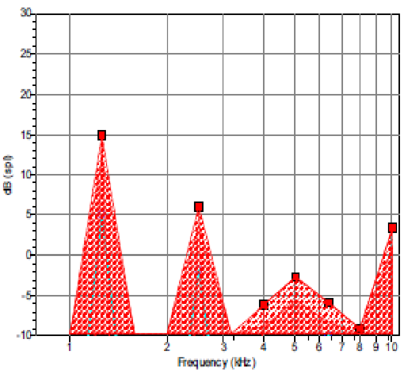

Test Summary
Sum all 1/2 octave = -27,0dBspl
Ave DP 1/2oct (1-6) = -36,0dBspl

Half octave band OAE power

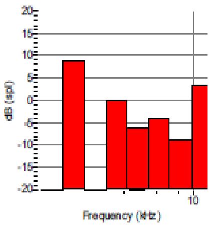

Freq (kHz)	Signal (dBspl)	Noise (dBspl)	SNR (dB)
1.0	-30,0	-100,0	70,0
1.4	-0,9	8,9	-9,7
2.0	-30,0	-100,0	70,0
2.8	-7,5	0,1	-7,6
4.0	-25,0	-6,1	-18,9
6.0	-13,4	-4,1	-9,3
8.0	-12,7	-9,0	-3,7
10.0	-13,7	3,5	-17,2

Test Environment
NLo = 102 NHi = 746 RejLev = 6mPa, 49,5dBspl Test time = 84s
Hardware= USBOAE Probe = Probe 1

Left ear

Ear canal frequency response

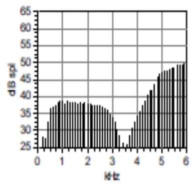

DPOAE response

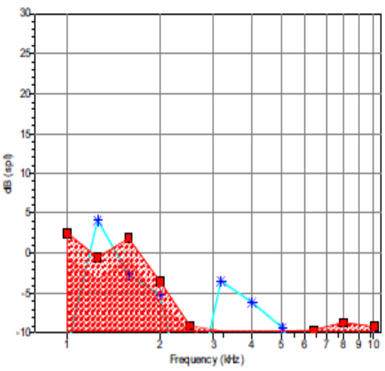

Test Summary
Sum all 1/2 octave = 2,4dBspl
Ave DP 1/2oct (1-6) = -6,6dBspl

Half octave band OAE power

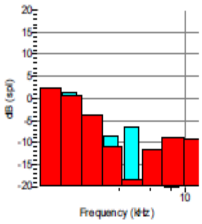

Freq (kHz)	Signal (dBspl)	Noise (dBspl)	SNR (dB)
1.0	-11,8	2,5	-14,3
1.4	1,3	0,7	0,6
2.0	-5,2	-3,5	-1,7
2.8	-8,4	-10,8	2,4
4.0	-6,2	-18,3	12,1
6.0	-12,4	-11,6	-0,8
8.0	-22,4	-8,7	-13,7
10.0	-10,5	-9,2	-1,3

Test Environment
NLo = 539 NHi = 117 RejLev = 6mPa, 49,5dBspl Test time = 63s
Hardware= USBOAE Probe = Probe 1

Right

Freq Hz	L1 dBspl	L2 dBspl	DP dBspl	2SD Noise dBspl	1SD Noise dBspl	SNR dB
1001	0,0	0,0	-30,0	-100,0	-100,0	70,0
1257	64,1	55,5	5,0	14,9	12,2	-9,9
1587	0,0	0,0	-30,0	-100,0	-100,0	70,0
2002	0,0	0,0	-30,0	-100,0	-100,0	70,0
2515	64,3	51,2	-1,8	6,1	3,8	-7,9
3174	65,0 *	55,0 *	-30,0	-100,0	-100,0	70,0
4004	65,0 *	55,3	-25,0	-6,1	-8,8	-18,9
5042	66,1	57,8	-18,9	-2,6	-6,6	-16,3
6348	67,7	55,0 *	-10,0	-5,8	-8,3	-4,2
7996	67,9	55,0 *	-12,7	-9,0	-11,1	-3,7
10083	66,3	55,0 *	-13,7	3,5	1,1	-17,2

Left

Freq Hz	L1 dBspl	L2 dBspl	DP dBspl	2SD Noise dBspl	1SD Noise dBspl	SNR dB
1001	65,5	54,3	-11,8	2,5	-0,5	-14,3
1257	64,6	55,0	4,1	-0,5	-3,3	4,6
1587	65,4	54,7	-2,7	1,8	-0,7	-4,5
2002	65,3	54,8	-5,2	-3,5	-6,3	-1,7
2515	65,3	54,2	-20,9	-9,0	-11,4	-11,9
3174	64,0	55,0 *	-3,5	-13,1	-15,6	9,6
4004	65,0 *	53,5	-6,2	-18,3	-20,6	12,1
5042	63,7	57,0	-9,4	-14,2	-17,0	4,8
6348	67,4	56,8	-17,0	-9,6	-12,4	-7,4
7996	67,2	55,0 *	-22,4	-8,7	-11,8	-13,7
10083	67,5	55,0 *	-10,5	-9,2	-11,8	-1,3

Supplement 1. Audiological assessments per patient

TEOAE Test Report – PID 04

Right

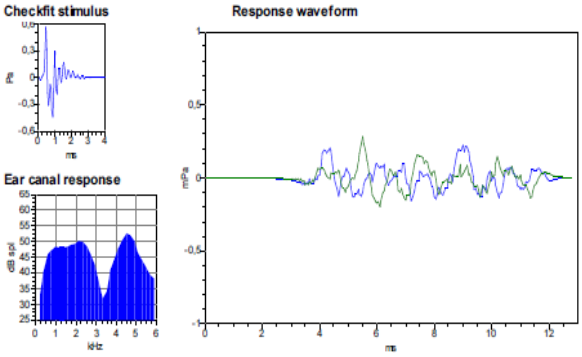

Half octave band OAE power

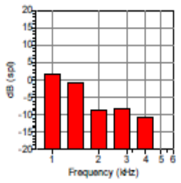

Freq (kHz)	Signal (dB spl)	Noise (dBspl)	SNR (dB)
1.0	-5,5	1,8	-7,3
1.4	-10,7	-1,1	-9,6
2.0	-19,2	-9,0	-10,2
2.8	-30,0	-8,5	-21,5
4.0	-16,9	-10,8	-6,1

Test Summary

Total OAE response = -50,0dBspl   Total Noise =6,9dBspl

Test Environment

NLo = 157   NHi = 317   RejLev = 6mPa, 49,5dBspl   Repro = -3%   Stim stab = 98%

Test time = 81s   Hardware= USBOAE   Probe = Probe 1

Left

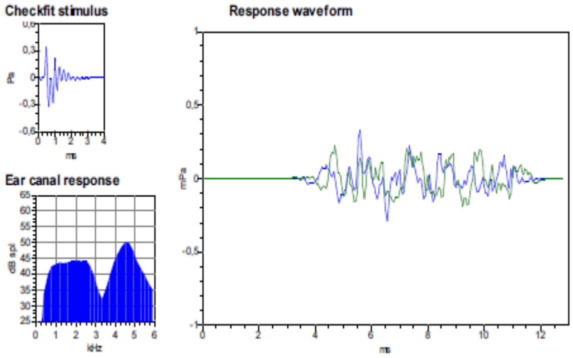

Half octave band OAE power

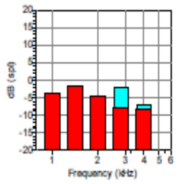

Freq (kHz)	Signal (dB spl)	Noise (dBspl)	SNR (dB)
1.0	-6,9	-3,7	-3,3
1.4	-15,0	-1,7	-13,3
2.0	-7,8	-4,7	-3,1
2.8	-2,0	-8,0	5,9
4.0	-6,9	-8,3	1,4

Test Summary

Total OAE response = -0,8dBspl   Total Noise =6,3dBspl

Test Environment

NLo = 208   NHi = 294   RejLev = 6mPa, 49,5dBspl   Repro =23%   Stim stab = 98%

Test time = 91s   Hardware= USBOAE   Probe = Probe 1

Supplement 1. Audiological assessments per patient

Tympanometry – PID 04

Tympanogram (Tymp)

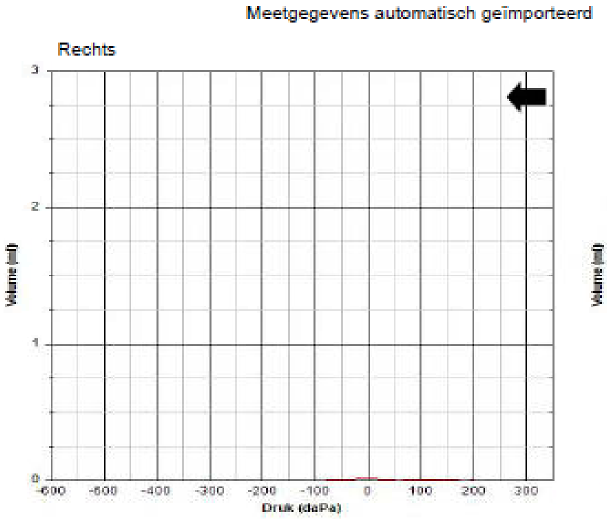

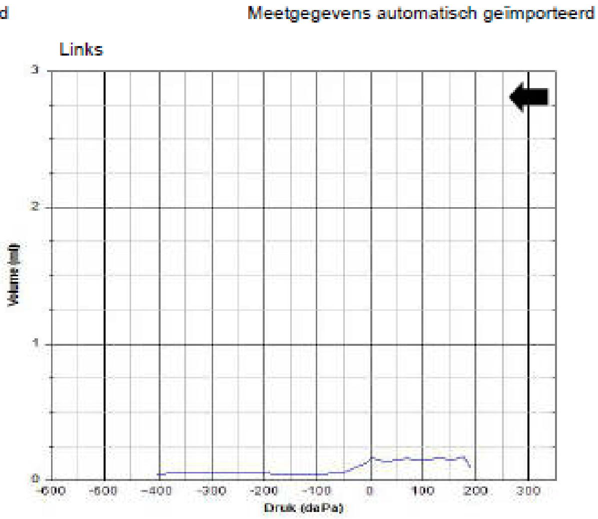

ECV.	0.54 ml	Begin druk	200.00 daPa
Compliantie	0.01 ml	Eind druk	-400.00 daPa
Druk	4.00 daPa	Pomp snelheid	Maximaal
Gradiënt	NaN ml	Test toon	226 Hz

ECV.	0.46 ml	Begin druk	200.00 daPa
Compliantie	0.17 ml	Eind druk	-400.00 daPa
Druk	180.00 daPa	Pomp snelheid	Maximaal
Gradiënt	NaN ml	Test toon	226 Hz

Patient ID: 05

Sex: female

Age: 23 months

DP-OAE Test Report – PID 05

Right ear

Ear canal frequency response

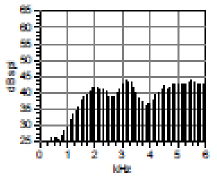

DPOAE response

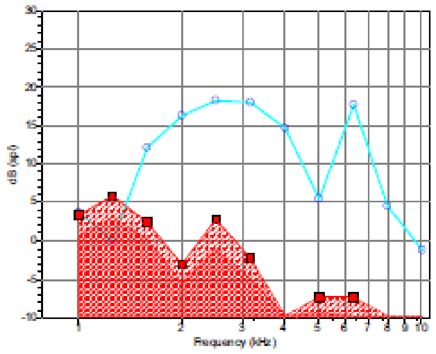

Test Summary
Sum all 1/2 octave = 22,4dBspl
Ave DP 1/2oct (1-6) = 13,3dBspl

Half octave band OAE power

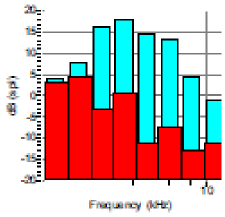

Freq (kHz)	Signal (dBspl)	Noise (dBspl)	SNR (dB)
1.0	3,8	3,3	0,5
1.4	8,0	4,3	3,7
2.0	16,3	-3,1	19,4
2.8	18,2	0,6	17,5
4.0	14,7	-11,4	26,1
6.0	13,6	-7,3	20,9
8.0	4,5	-12,8	17,3
10.0	-1,2	-11,4	10,2

Test Environment
NLo = 564 NHi = 268 RejLev = 6mPa, 49,5dBspl Testtime = 79s
Hardware= USBOAE Probe = Probe 1

Left ear

Ear canal frequency response

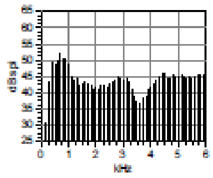

DPOAE response

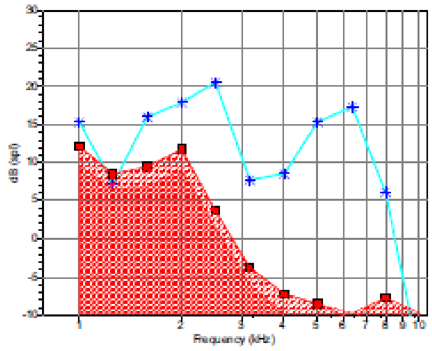

Test Summary
Sum all 1/2 octave = 23,2dBspl
Ave DP 1/2oct (1-6) = 14,2dBspl

Half octave band OAE power

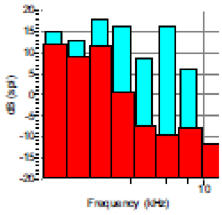

Freq (kHz)	Signal (dBspl)	Noise (dBspl)	SNR (dB)
1.0	15,3	12,1	3,2
1.4	12,7	9,1	3,6
2.0	17,8	11,8	6,0
2.8	16,4	0,8	15,5
4.0	8,6	-7,2	15,8
6.0	16,4	-9,5	25,9
8.0	6,1	-7,7	13,8
10.0	-16,4	-11,7	-4,7

Test Environment
NLo = 457 NHi = 647 RejLev = 8mPa, 52,0dBspl Testtime = 105s
Hardware= USBOAE Probe = Probe 1

Right

Freq Hz	L1 dBspl	L2 dBspl	DP dBspl	2SD Noise dBspl	1SD Noise dBspl	SNR dB
1001	55,8	49,3	3,8	3,3	0,7	0,5
1257	60,2	53,2	0,0	5,9	3,0	-5,9
1587	64,5	55,2	12,1	2,4	0,2	9,7
2002	65,8	55,1	16,3	-3,1	-5,4	19,4
2515	64,6	51,9	18,3	2,8	-1,1	15,5
3174	62,2	54,5	18,0	-2,3	-5,0	20,3
4004	63,3	55,0 *	14,7	-11,4	-13,6	26,1
5042	62,2	56,2	5,5	-7,3	-9,4	12,8
6348	67,1	57,0	17,7	-7,3	-9,8	25,0
7996	66,7	57,5	4,5	-12,8	-14,6	17,3
10083	67,3	55,0 *	-1,2	-11,4	-13,9	10,2

Left

Freq Hz	L1 dBspl	L2 dBspl	DP dBspl	2SD Noise dBspl	1SD Noise dBspl	SNR dB
1001	65,0 *	55,0 *	15,3	12,1	9,6	3,2
1257	65,0 *	55,0 *	7,2	8,6	6,5	-1,4
1587	65,0 *	54,0	16,0	9,5	6,8	6,5
2002	64,3	54,3	17,8	11,8	9,1	6,0
2515	64,9	55,5	20,6	3,8	1,5	16,8
3174	65,8	52,7	7,8	-3,7	-6,2	11,5
4004	62,3	56,8	8,6	-7,2	-9,7	15,8
5042	67,9	57,9	15,3	-8,5	-11,0	23,8
6348	67,3	55,0 *	17,4	-10,7	-13,6	28,1
7996	65,0 *	55,0 *	6,1	-7,7	-10,4	13,8
10083	67,8	55,0 *	-16,4	-11,7	-13,4	-4,7

TEOAE Test Report – PID 05

Right

Test type: TE - QuickScreen
Stimulus: 84,0dB peSPL
Mode: Gen Diag
Tester ID: AUD
Data file: 1ROUBI30.DTA
Notes:

Response waveform

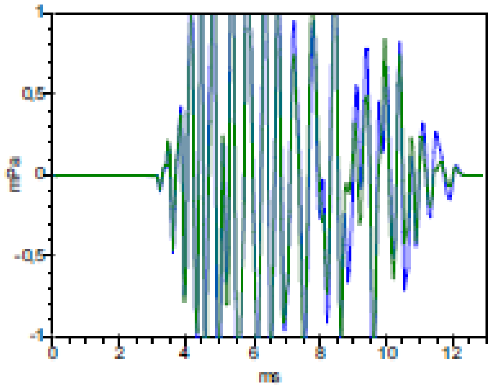

Half octave band OAE power

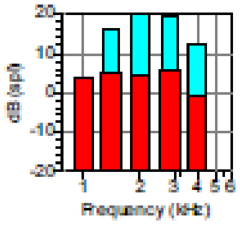

Freq (kHz)	Signal (dB spl)	Noise (dB spl)	SNR (dB)
1.0	3,0	3,6	-0,7
1.4	15,9	4,9	11,0
2.0	21,5	3,9	17,5
2.8	19,7	5,3	14,4
4.0	11,7	-0,8	12,5

Test Summary
Total OAEresponse = 24,6dBspl Total Noise = 11,3dBspl

Checkfit stimulus

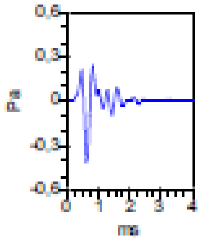

Ear canal response

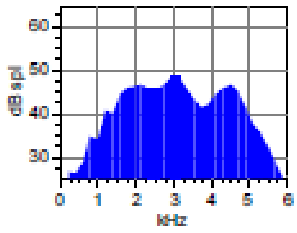

Test Environment

NLo = 199 NHi = 136 Test time = 69s
RejLev = 52,0dBspl Repro = 95% Stim stab = 99%
Hardware=USBOAE Probe = Probe 1

Left

Test type: TE - QuickScreen
Stimulus: 84,0dB peSPL
Mode: Gen Diag
Tester ID: AUD
Data file: 1ROUBI32.DTA
Notes:

Response waveform

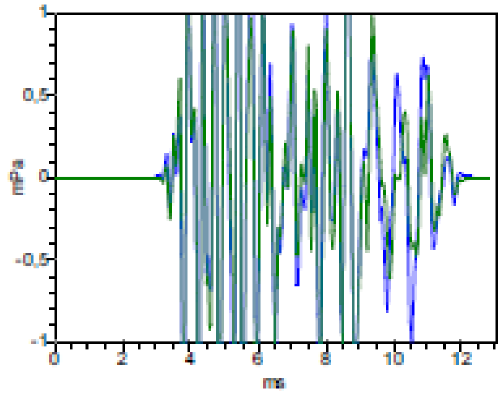

Half octave band OAE power

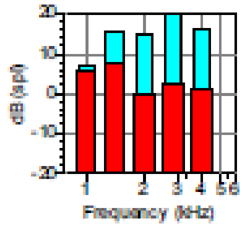

Freq (kHz)	Signal (dB spl)	Noise (dB spl)	SNR (dB)
1.0	7,1	5,4	1,7
1.4	14,9	7,4	7,5
2.0	14,9	-0,2	15,1
2.8	22,8	2,3	20,5
4.0	16,0	1,0	15,0

Test Summary
Total OAEresponse = 24,7dBspl Total Noise = 12,1dBspl

Checkfit stimulus

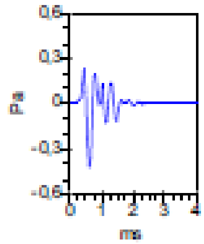

Ear canal response

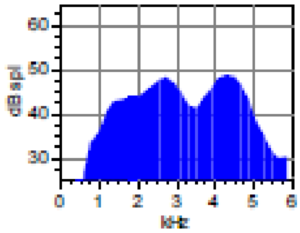

Test Environment

NLo = 156 NHi = 165 Test time = 62s
RejLev = 52,0dBspl Repro = 95% Stim stab = 98%
Hardware= USBOAE Probe = Probe 1

Supplement 1. Audiological assessments per patient

Tympanometry – PID 05

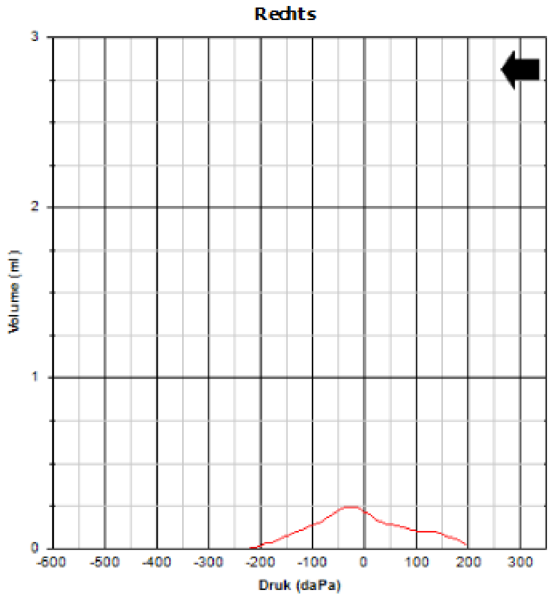

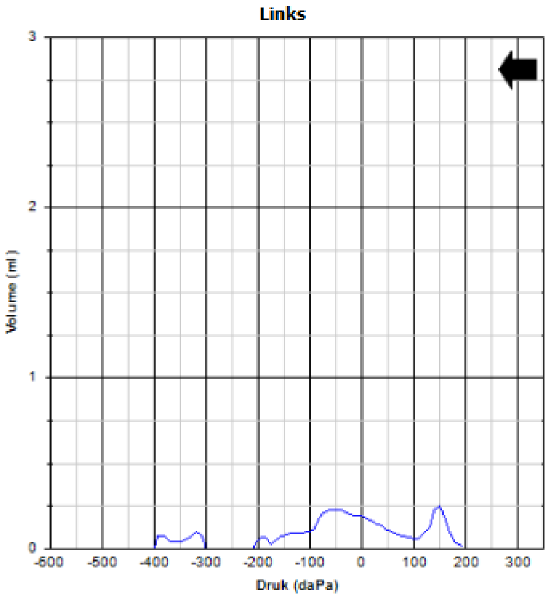

**ECV.**	0,43 ml	**Begin druk**	200,00 daPa
**Compliantie**	0,24 ml	**Eind druk**	-400,00 daPa
**Druk**	-28,00 daPa	**Pomp snelheid**	Maximaal
**Gradiënt**	0,08 ml	**Test toon**	226 Hz

**ECV.**	0,45 ml	**Begin druk**	200,00 daPa
**Compliantie**	0,25 ml	**Eind druk**	-400,00 daPa
**Druk**	151,00 daPa	**Pomp snelheid**	Maximaal
**Gradiënt**	0,22 ml	**Test toon**	226 Hz

Patient ID: 06

Sex: female

Age: 20 months

Supplement 1. Audiological assessments per patient

DP-OAE Test Report – PID 06

Right ear

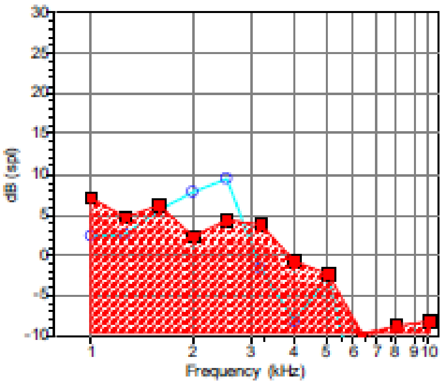

Half octave band OAE power

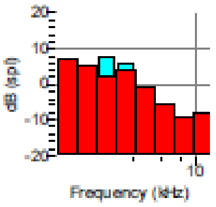

Freq (kHz)	Signal (dB spl)	Noise (dB spl)	SNR (dB)
1.0	2,4	7,1	-4,7
1.4	4,2	5,5	-1,2
2.0	7,9	2,4	5,5
2.8	5,5	4,1	1,5
4.0	-8,2	-0,8	-7,4
6.0	-7,4	-5,5	-1,9
8.0	-16,6	-8,8	-7,8
10.0	-12,6	-8,2	-4,4

Test Summary

Sum all 1/2 octave = 9,9dBspl   Ave DP 1/2oct (1-6) =0,9dBspl

Ear canal frequency response

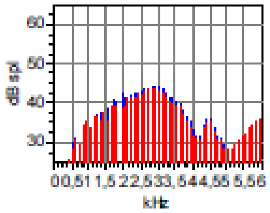

Test Environment

NLo = 356   NHi = 828   Test time = 112s
RejLev = 52,0dBspl
Hardware:USBOAE   Probe = Probe 1

Left ear

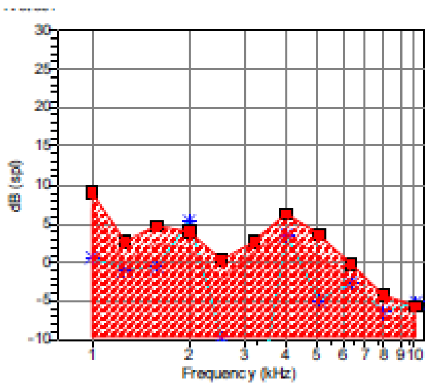

Half octave band OAE power

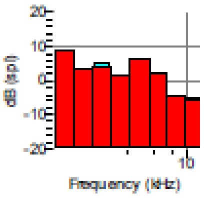

Freq (kHz)	Signal (dB spl)	Noise (dB spl)	SNR (dB)
1.0	0,8	9,1	-8,3
1.4	-0,7	3,8	-4,6
2.0	5,4	4,0	1,4
2.8	-13,6	1,7	-15,3
4.0	3,7	6,4	-2,7
6.0	-3,6	1,9	-5,5
8.0	-6,4	-4,1	-2,3
10.0	-5,1	-5,7	0,6

Test Summary

Sum all 1/2 octave = 5,8dBspl   Ave DP 1/2oct (1-6) =-3,3dBspl

Ear canal frequency response

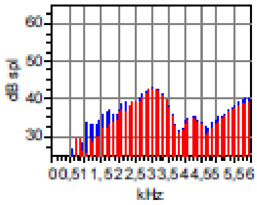

Test Environment

NLo = 241   NHi = 1151   Test time = 132s
RejLev = 52,0dBspl
Hardware:USBOAE   Probe = Probe 1

Left

Freq Hz	L1 dBspl	L2 dBspl	DP dBspl	2SD Noise dBspl	1SD Noise dBspl	SNR dB
1001	65,0 *	55,0 *	0,8	9,1	6,9	-8,3
1257	65,0 *	55,0 *	-0,9	2,7	0,0	-3,6
1587	65,0 *	55,0 *	-0,6	4,8	2,6	-5,4
2002	65,0 *	53,2	5,4	4,0	1,7	1,4
2515	62,8	52,9	-9,6	0,4	-2,1	-10,0
3174	63,8	53,0	-21,1	2,8	1,4	-23,9
4004	63,7	52,1	3,7	6,4	3,9	-2,7
5042	63,0	53,9	-4,9	3,6	0,6	-8,5
6348	66,0	54,9	-2,5	-0,2	-2,3	-2,3
7996	66,1	55,1	-6,4	-4,1	-6,8	-2,3
10083	65,3	57,7	-5,1	-5,7	-7,8	0,6

Right

Freq Hz	L1 dBspl	L2 dBspl	DP dBspl	2SD Noise dBspl	1SD Noise dBspl	SNR dB
1001	57,8	50,5	2,4	7,1	5,2	-4,7
1257	62,9	51,5	2,6	4,8	2,4	-2,2
1587	64,4	50,9	5,6	6,1	3,7	-0,5
2002	66,5	54,2	7,9	2,4	-0,1	5,5
2515	65,7	54,0	9,4	4,3	1,7	5,1
3174	66,4	55,0 *	-1,6	3,8	1,5	-5,4
4004	65,6	55,0 *	-8,2	-0,8	-3,2	-7,4
5042	65,0 *	55,0 *	-2,3	-2,4	-4,0	0,1
6348	65,0 *	54,3	-21,2	-10,2	-12,9	-11,0
7996	67,0	54,4	-16,6	-8,8	-11,3	-7,8
10083	67,6	57,4	-12,6	-8,2	-10,5	-4,4

Supplement 1. Audiological assessments per patient

TEOAE Test Report – PID 06

Right

Test type: TE - Quick Screen
Stimulus: 84,4 dB peSPL
Mode: Gen Diag
Tester ID: AUD
Data file: 1ROV2H30.DTA
Notes:

Response waveform

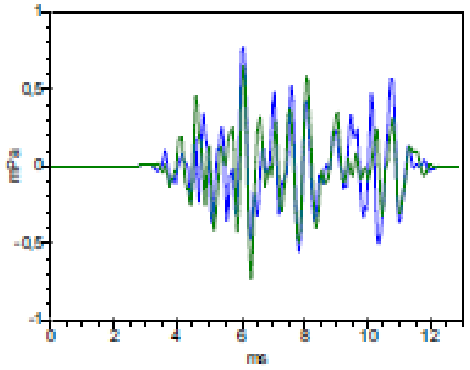

Half octave band OAE power

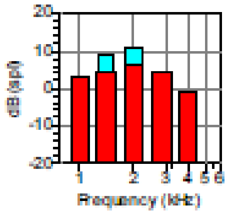

Freq (kHz)	Signal (dB spl)	Noise (dBspl)	SNR (dB)
1.0	-1,9	2,7	-4,5
1.4	9,1	4,3	4,8
2.0	10,5	6,0	4,5
2.8	3,5	4,6	-1,1
4.0	-1,8	-0,9	-0,9

Test Summary
Total OAE response = 12,9dBspl Total Noise = 11,2dBspl

Checkfit stimulus

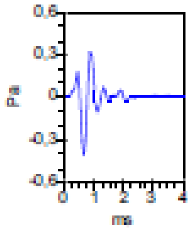

Ear canal response

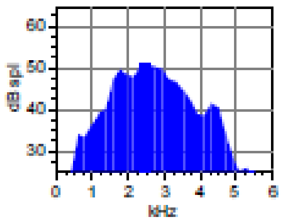

Test Environment
NLo = 260 NH = 440 Test time = 122s
RejLev = 52,0dBspl Repro = 64% Stim stab = 99%
Hardware: USBOAE Probe = Probe 1

Left

Test type: TE - Quick Screen
Stimulus: 87,0dB peSPL
Mode: Gen Diag
Tester ID: AUD
Data file: 1ROV2H32.DTA
Notes:

Response waveform

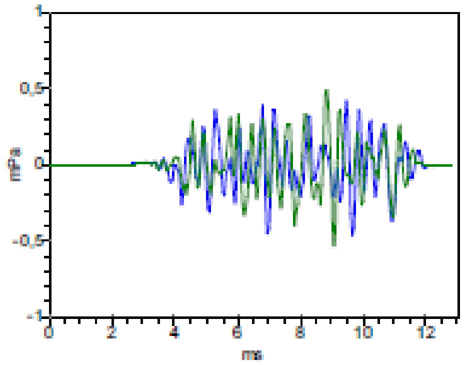

Half octave band OAE power

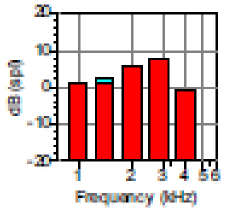

Freq (kHz)	Signal (dBspl)	Noise (dBspl)	SNR (dB)
1.0	-11,1	0,9	-12,0
1.4	2,2	1,2	1,0
2.0	4,5	5,8	-1,3
2.8	5,1	7,2	-2,0
4.0	-3,9	-0,8	-3,1

Test Summary
Total OAE response = 2,2dBspl Total Noise = 11,2dBspl

Checkfit stimulus

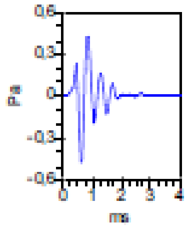

Ear canal response

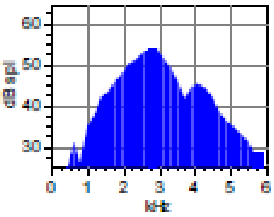

Test Environment
NLo = 260 NH = 204 Test time = 93s
RejLev = 52,0dBspl Repro = 35% Stim stab = 98%
Hardware = USBOAE Probe = Probe 1

Tympanometry – PID 06

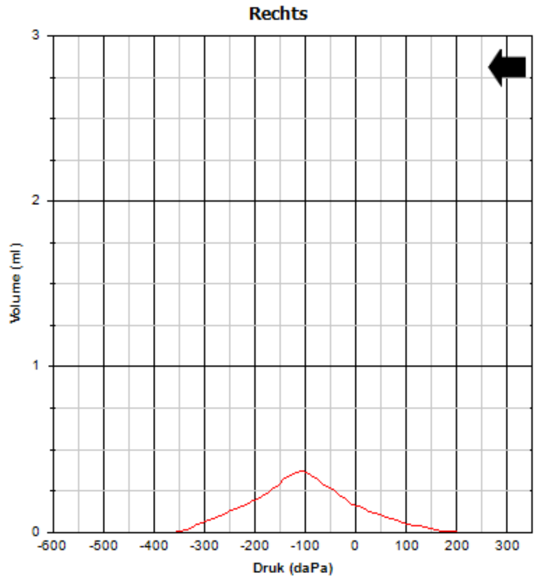

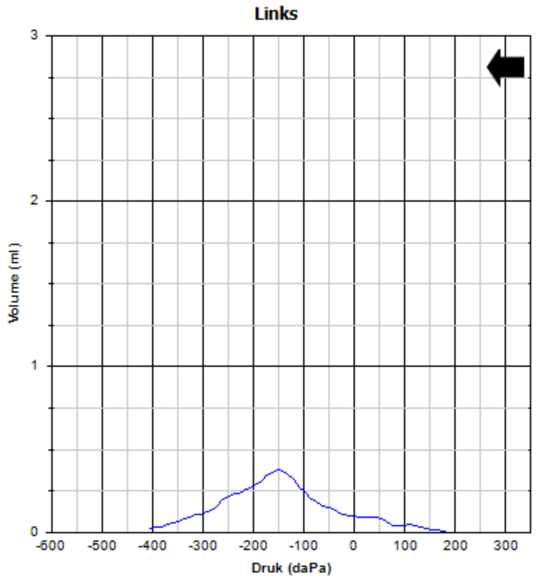

**ECV.**	0,42 ml	**Begin druk**	200,00 daPa
**Compliantie**	0,36 ml	**Eind druk**	-400,00 daPa
**Druk**	-106,00 daPa	**Pomp snelheid**	Maximaal
**Gradiënt**	0,09 ml	**Test toon**	226 Hz

**ECV.**	0,42 ml	**Begin druk**	200,00 daPa
**Compliantie**	0,37 ml	**Eind druk**	-400,00 daPa
**Druk**	-150,00 daPa	**Pomp snelheid**	Maximaal
**Gradiënt**	0,11 ml	**Test toon**	226 Hz

Patient ID: 07

Sex: female

Age: 20 months

Supplement 1. Audiological assessments per patient

DP-OAE Test Report – PID 07

Right ear

Ear canal frequency response

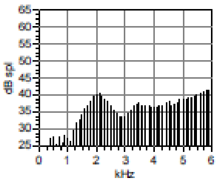

DPOAE response

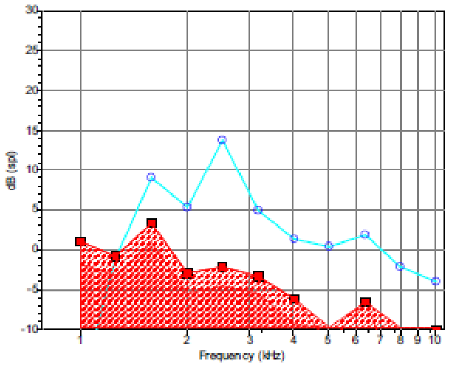

Test Summary
Sum all 1/2 octave = 13,3dBspl
Ave DP 1/2oct (1-6) = 4,3dBspl

Half octave band OAE power

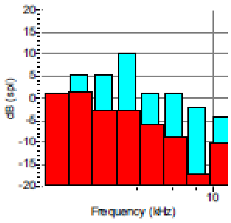

Freq (kHz)	Signal (dBspl)	Noise (dBspl)	SNR (dB)
1.0	-17,4	1,0	-18,4
1.4	5,4	1,5	3,9
2.0	5,3	-3,0	8,3
2.8	10,4	-2,7	13,1
4.0	1,3	-6,1	7,4
6.0	1,1	-8,7	9,8
8.0	-2,2	-17,3	15,1
10.0	-4,0	-10,1	6,1

Test Environment
NLo = 772 NHi = 364 RejLev = 8mPa, 52,0dBspl Test time = 111s
Hardware= USBOAE Probe = Probe 1

Left ear

Ear canal frequency response

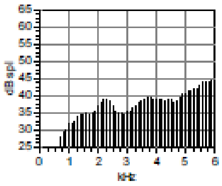

DPOAE response

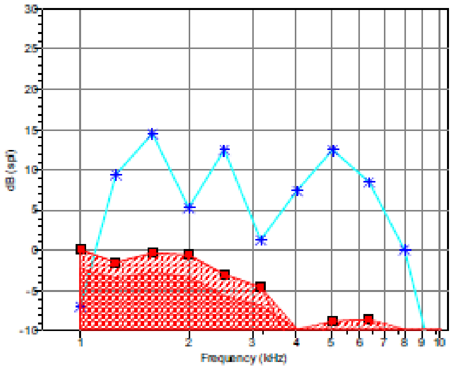

Test Summary
Sum all 1/2 octave = 16,7dBspl
Ave DP 1/2oct (1-6) = 7,6dBspl

Half octave band OAE power

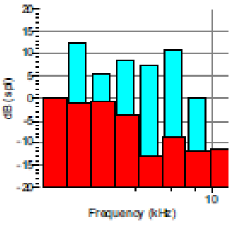

Freq (kHz)	Signal (dBspl)	Noise (dBspl)	SNR (dB)
1.0	-6,9	0,1	-7,0
1.4	12,4	-0,9	13,2
2.0	5,4	-0,6	6,0
2.8	8,7	-3,7	12,4
4.0	7,4	-13,1	20,5
6.0	10,7	-8,6	19,4
8.0	0,1	-11,7	11,8
10.0	-17,7	-11,3	-6,4

Test Environment
NLo = 1000 NHi = 72 RejLev = 8mPa, 52,0dBspl Test time = 102s
Hardware= USBOAE Probe = Probe 1

Right

Freq Hz	L1 dBspl	L2 dBspl	DP dBspl	2SD Noise dBspl	1SD Noise dBspl	SNR dB
1001	53,1	46,7	-17,4	1,0	-1,8	-18,4
1257	57,8	51,5	-1,0	-0,8	-3,1	-0,2
1587	62,5	55,0	9,0	3,3	0,9	5,7
2002	65,8	55,1	5,3	-3,0	-5,5	8,3
2515	64,9	49,6	13,7	-2,2	-4,5	15,9
3174	65,0 *	55,0 *	4,9	-3,3	-5,5	8,2
4004	65,0 *	55,0 *	1,3	-6,1	-9,4	7,4
5042	65,0 *	54,9	0,4	-11,7	-13,6	12,1
6348	65,8	57,5	1,8	-6,5	-10,2	8,3
7996	67,7	57,0	-2,2	-17,3	-19,8	15,1
10083	66,3	55,0 *	-4,0	-10,1	-12,6	6,1

Left

Freq Hz	L1 dBspl	L2 dBspl	DP dBspl	2SD Noise dBspl	1SD Noise dBspl	SNR dB
1001	62,6	53,7	-6,9	0,1	-2,2	-7,0
1257	64,1	52,7	9,5	-1,5	-3,3	11,0
1587	64,7	52,8	14,5	-0,3	-2,7	14,8
2002	63,8	53,9	5,4	-0,6	-3,2	6,0
2515	65,4	55,0 *	12,6	-2,9	-5,7	15,5
3174	62,3	52,7	1,3	-4,6	-6,8	5,9
4004	65,3	53,0	7,4	-13,1	-15,9	20,5
5042	64,7	55,2	12,5	-8,8	-11,4	21,3
6348	66,6	55,0	8,5	-8,5	-11,0	17,0
7996	66,8	55,7	0,1	-11,7	-13,6	11,8
10083	66,1	55,0 *	-17,7	-11,3	-13,1	-6,4

Supplement 1. Audiological assessments per patient

TEOAE Test Report – PID 07

Right

Checkfit stimulus

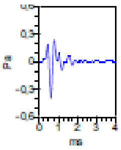

Response waveform

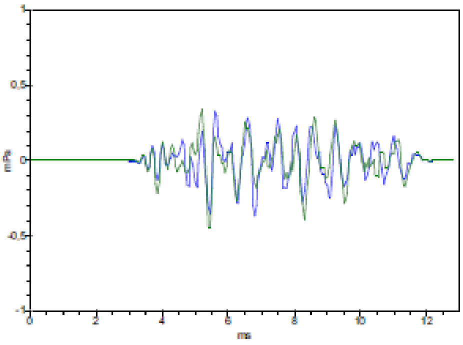

Ear canal response

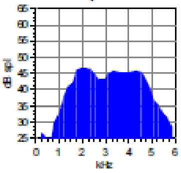

Half octave band OAE power

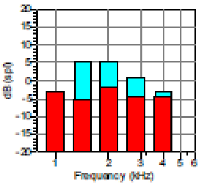

Freq (kHz)	Signal (dB spl)	Noise (dBspl)	SNR (dB)
1.0	-8,6	-3,2	-5,4
1.4	5,3	-5,2	10,5
2.0	5,5	-1,6	7,1
2.8	0,9	-4,4	5,3
4.0	-3,1	-4,2	1,2

Test Summary

Total OAE response = 9,4dBspl   Total Noise = 5,3dBspl

Test Environment

NLo = 260   NHi = 51   RejLev = 8mPa, 52,0dBspl   Repro = 71%   Stim stab = 99%

Test time = 74s   Hardware= USBOAE   Probe = Probe 1

Left

Checkfit stimulus

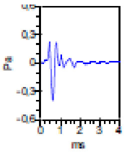

Response waveform

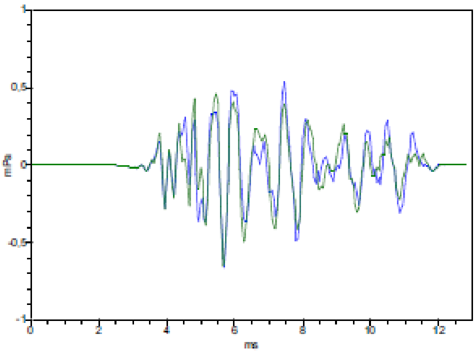

Ear canal response

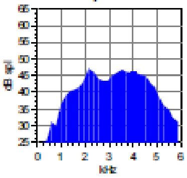

Half octave band OAE power

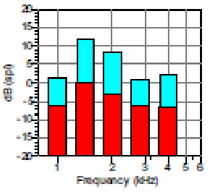

Freq (kHz)	Signal (dB spl)	Noise (dBspl)	SNR (dB)
1.0	1,5	-6,3	7,8
1.4	11,6	0,0	11,6
2.0	8,5	-2,9	11,4
2.8	0,7	-5,9	6,7
4.0	2,2	-6,8	9,0

Test Summary

Total OAE response = 14,1dBspl   Total Noise = 5,2dBspl

Test Environment

NLo = 260   NHi = 50   RejLev = 8mPa, 52,0dBspl   Repro = 89%   Stim stab = 99%

Test time = 74s   Hardware= USBOAE   Probe = Probe 1

Supplement 1. Audiological assessments per patient

Tympanometry – PID 07

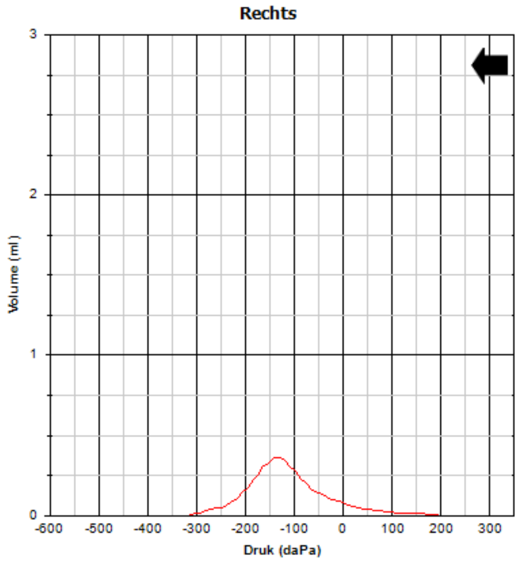

**ECV.**	0,62 ml	**Begin druk**	200,00 daPa
**Compliantie**	0,36 ml	**Eind druk**	-400,00 daPa
**Druk**	-128,00 daPa	**Pomp snelheid**	Maximaal
**Gradiënt**	0,14 ml	**Test toon**	226 Hz

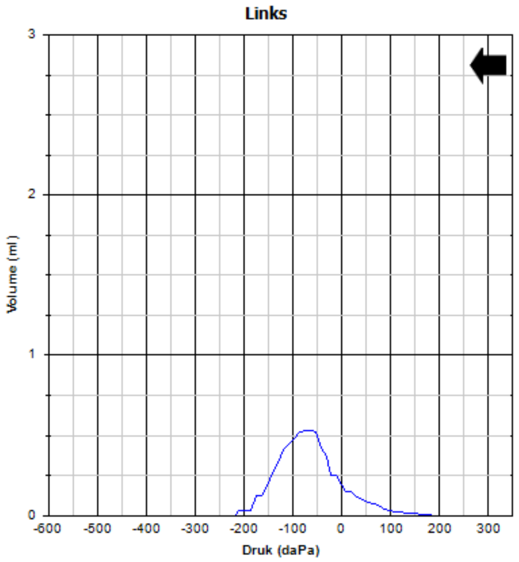

**ECV.**	0,59 ml	**Begin druk**	200,00 daPa
**Compliantie**	32,70 ml	**Eind druk**	-400,00 daPa
**Druk**	0,00 daPa	**Pomp snelheid**	Maximaal
**Gradiënt**	32,70 ml	**Test toon**	226 Hz

Patient ID: 08

Sex: female

Age: 20 months

DP-OAE Test Report – PID 08

Right

Ear canal frequency response

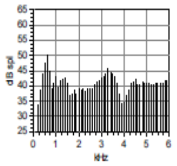

DPOAE response

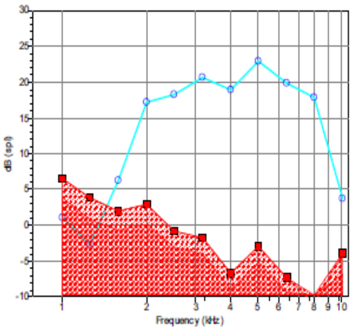

Test Summary
Sum all 1/2 octave = 26,3dBspl
Ave DP 1/2oct (1-6) = 17,3dBspl

Half octave band OAE power

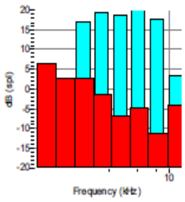

Freq (kHz)	Signal (dBspl)	Noise (dBspl)	SNR (dB)
1.0	1,0	6,5	-5,5
1.4	2,9	3,0	-0,1
2.0	17,2	3,0	14,2
2.8	19,6	-1,2	20,8
4.0	18,9	-6,7	25,6
6.0	21,5	-4,9	26,4
8.0	17,9	-11,0	28,9
10.0	3,7	-3,9	7,6

Test Environment
NLo = 455 NHi = 761 RejLev = 8mPa, 52,0dBspl Test time = 115s
Hardware= USBOAE Probe = Probe 1

Left

Ear canal frequency response

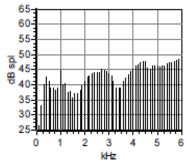

DPOAE response

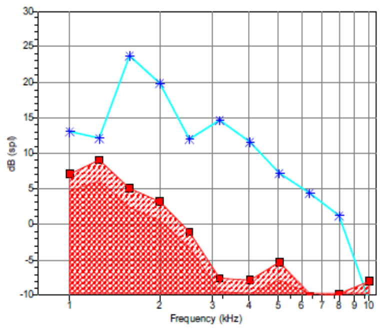

Test Summary
Sum all 1/2 octave = 24,1dBspl
Ave DP 1/2oct (1-6) = 15,0dBspl

Half octave band OAE power

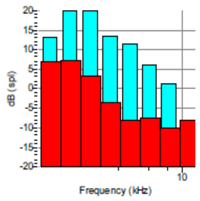

Freq (kHz)	Signal (dBspl)	Noise (dBspl)	SNR (dB)
1.0	13,1	7,1	6,0
1.4	19,8	7,3	12,5
2.0	19,9	3,2	16,7
2.8	13,5	-3,7	17,1
4.0	11,6	-7,9	19,5
6.0	5,9	-7,5	13,4
8.0	1,2	-9,9	11,1
10.0	-10,9	-8,0	-2,9

Test Environment
NLo = 466 NHi = 766 RejLev = 8mPa, 52,0dBspl Test time = 124s
Hardware= USBOAE Probe = Probe 1

Right

Freq Hz	L1 dBspl	L2 dBspl	DP dBspl	2SD Noise dBspl	1SD Noise dBspl	SNR dB
1001	50,8	50,6	1,0	6,5	3,8	-5,5
1257	52,6	53,5	-2,7	3,9	0,8	-6,6
1587	57,6	54,0	6,3	2,0	-0,6	4,3
2002	66,5	55,9	17,2	3,0	0,1	14,2
2515	66,2	55,1	18,3	-0,8	-3,1	19,1
3174	66,8	55,5	20,7	-1,7	-4,0	22,4
4004	66,5	55,5	18,9	-6,7	-9,0	25,6
5042	67,1	56,8	22,9	-3,0	-5,2	25,9
6348	65,0 *	57,4	19,9	-7,3	-9,4	27,2
7996	65,0 *	55,0 *	17,9	-11,0	-13,3	28,9
10083	65,0 *	55,0	3,7	-3,9	-6,9	7,6

Left

Freq Hz	L1 dBspl	L2 dBspl	DP dBspl	2SD Noise dBspl	1SD Noise dBspl	SNR dB
1001	65,0 *	55,0 *	13,1	7,1	4,7	6,0
1257	65,0 *	55,0 *	12,1	9,0	6,1	3,1
1587	62,1	52,2	23,8	5,1	2,4	18,7
2002	63,9	54,0	19,9	3,2	0,5	16,7
2515	64,2	55,0 *	12,0	-1,0	-3,3	13,0
3174	63,9	55,0 *	14,7	-7,6	-9,4	22,3
4004	65,0 *	52,7	11,6	-7,9	-9,8	19,5
5042	65,5	54,1	7,2	-5,4	-7,9	12,6
6348	65,9	55,0	4,4	-10,2	-12,6	14,6
7996	66,9	55,0	1,2	-9,9	-12,1	11,1
10083	66,4	57,1	-10,9	-8,0	-10,4	-2,9

TEOAE Test Report – PID 08

Right

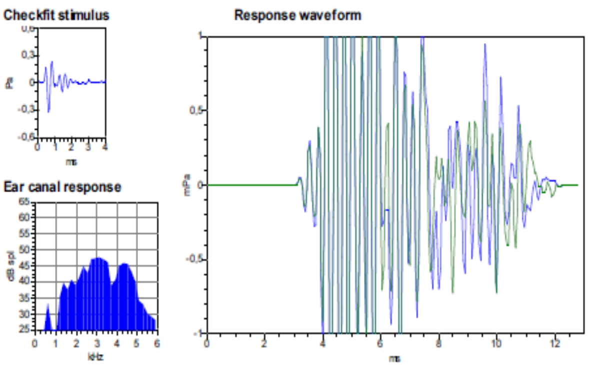

Half octave band OAE power

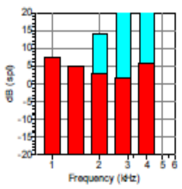

Freq (kHz)	Signal (dB spl)	Noise (dBspl)	SNR (dB)
1.0	-1,0	7,3	-8,3
1.4	3,0	4,7	-1,8
2.0	14,1	2,8	11,3
2.8	20,5	1,6	18,9
4.0	21,5	5,6	15,9

Test Summary
Total OAE response = 24,5dBspl Total Noise =13,2dBspl

Test Environment
NLo = 204 NHi = 1352 RejLev = 8mPa, 52,0dBspl Repro =93% Stim stab = 98%
Test time = 222s Hardware= USBOAE Probe = Probe 1

Left

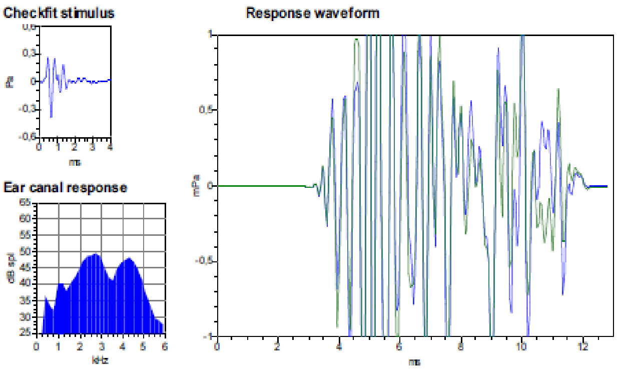

Half octave band OAE power

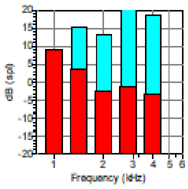

Freq (kHz)	Signal (dB spl)	Noise (dBspl)	SNR (dB)
1.0	7,9	9,3	-1,4
1.4	15,3	3,7	11,6
2.0	13,4	-2,6	16,0
2.8	23,8	-1,3	25,1
4.0	18,5	-3,1	21,6

Test Summary
Total OAE response = 25,7dBspl Total Noise =13,4dBspl

Test Environment
NLo = 190 NHi = 231 RejLev = 8mPa, 52,0dBspl Repro =94% Stim stab = 0%
Test time = 79s Hardware= USBOAE Probe = Probe 1

Tympanometry – PID 08

Tympanogram (Tymp)

Meetgegevens automatisch geïmporteerd

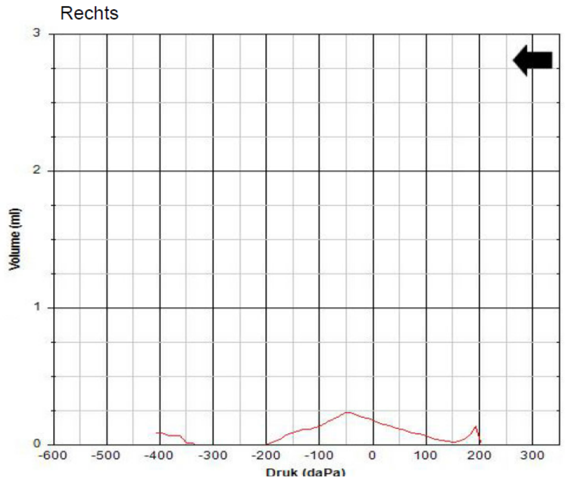

Meetgegevens automatisch geïmporteerd

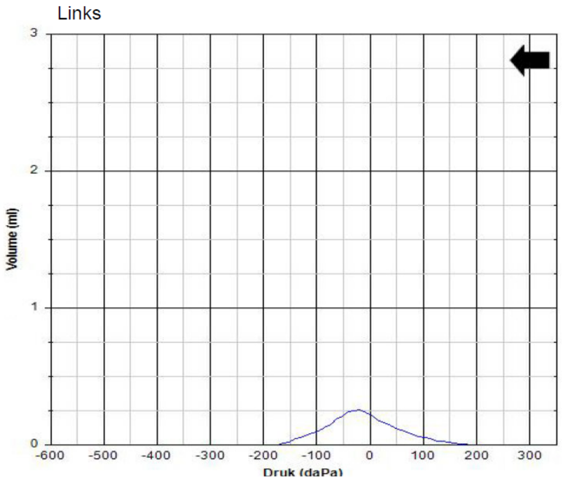

Patient ID: 09

Sex: male

Age: 19 months

DP-OAE Test Report – PID 09

Right ear

Not performed, not cooperative

Left ear

Ear canal frequency response

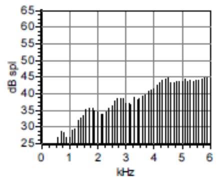

DPOAE response

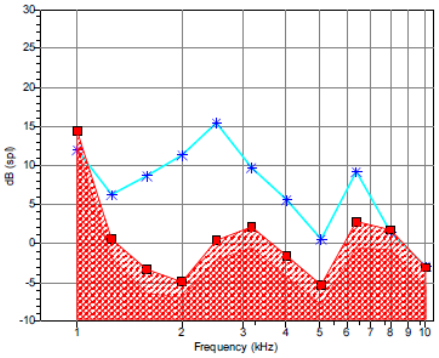

Test Summary
Sum all 1/2 octave = 16,7dBspl
Ave DP 1/2oct (1-6) = 7,7dBspl

Half octave band OAE power

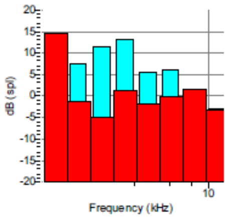

Freq (kHz)	Signal (dBspl)	Noise (dBspl)	SNR (dB)
1.0	12,0	14,4	-2,4
1.4	7,6	-1,1	8,7
2.0	11,3	-4,9	16,2
2.8	13,0	1,3	11,7
4.0	5,6	-1,6	7,2
6.0	5,9	-0,3	6,3
8.0	1,5	1,7	-0,2
10.0	-3,0	-3,1	0,1

Test Environment
NLo = 227 NHi = 557 RejLev = 8mPa, 52,0dBspl Test time = 76s
Hardware= USBOAE Probe = Probe 1

Left

Freq Hz	L1 dBspl	L2 dBspl	DP dBspl	2SD Noise dBspl	1SD Noise dBspl	SNR dB
1001	65,0 *	53,2	12,0	14,4	12,4	-2,4
1257	63,0	53,2	6,3	0,6	-1,9	5,7
1587	64,6	54,5	8,7	-3,3	-6,3	12,0
2002	65,6	54,0	11,3	-4,9	-6,9	16,2
2515	63,8	54,8	15,4	0,4	-2,1	15,0
3174	64,3	53,8	9,7	2,1	-0,5	7,6
4004	63,0	54,3	5,6	-1,6	-4,3	7,2
5042	64,0	55,4	0,6	-5,3	-7,9	5,9
6348	65,3	55,9	9,2	2,8	-0,5	6,4
7996	65,9	57,0	1,5	1,7	-0,8	-0,2
10083	66,3	55,0 *	-3,0	-3,1	-5,6	0,1

TEOAE Test Report – PID 09

Right ear

Checkfit stimulus

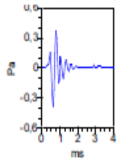

Response waveform

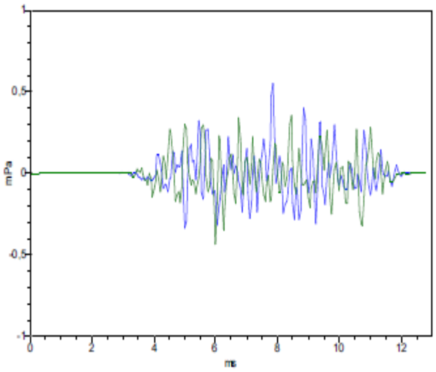

Ear canal response

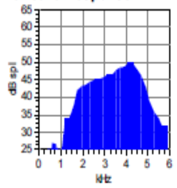

Half octave band OAE power

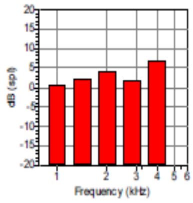

Freq (kHz)	Signal (dB spl)	Noise (dBspl)	SNR (dB)
1.0	-11,9	0,7	-12,6
1.4	-13,0	2,1	-15,1
2.0	-4,5	3,9	-8,4
2.8	-6,2	1,8	-7,9
4.0	1,8	6,8	-5,0

Test Summary

Total OAE response = -50,0dBspl Total Noise =11,6dBspl

Test Environment

NLo = 138 NHi = 143 RejLev = 8mPa, 52,0dBspl Repro = -9% Stim stab = 77%

Test time = 54s Hardware= USBOAE Probe = Probe 1

Left ear

Checkfit stimulus

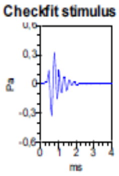

Response waveform

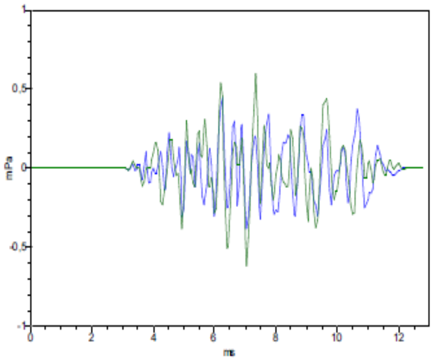

Ear canal response

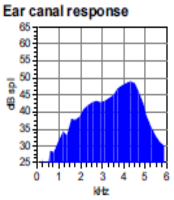

Half octave band OAE power

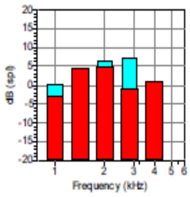

Freq (kHz)	Signal (dB spl)	Noise (dBspl)	SNR (dB)
1.0	0,2	-2,9	3,1
1.4	1,2	4,3	-3,1
2.0	6,6	4,7	1,9
2.8	7,0	-1,2	8,2
4.0	-1,3	0,9	-2,2

Test Summary

Total OAE response = 10,3dBspl Total Noise =10,2dBspl

Test Environment

NLo = 260 NHi = 105 RejLev = 8mPa, 52,0dBspl Repro = 54% Stim stab = 98%

Test time = 81s Hardware= USBOAE Probe = Probe 1

Tympanometry – PID 09

Tympanogram (Tymp)

Meetgegevens automatisch geïmporteerd

Rechts

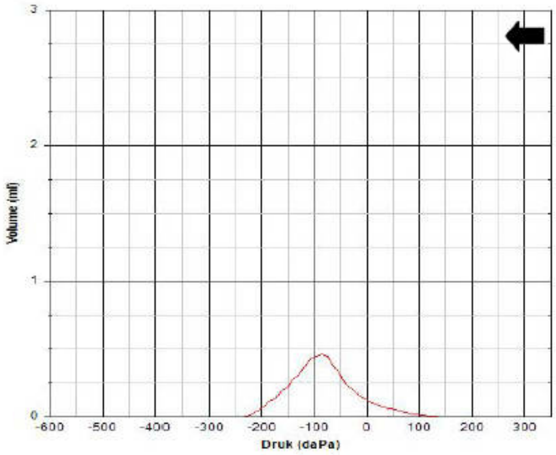

Meetgegevens automatisch geïmporteerd

Links

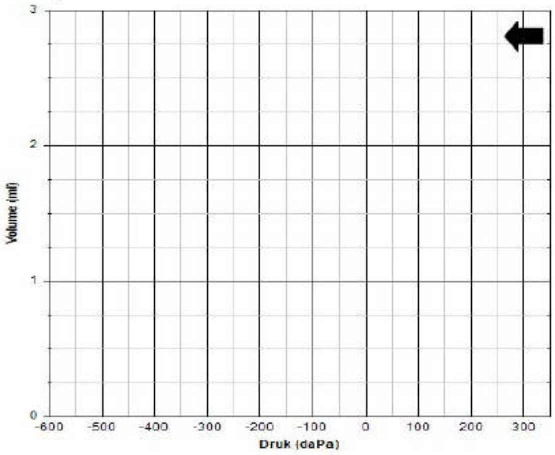

ECV.	0.52 ml	Begin druk	200.00 daPa
Compliantie	0.46 ml	Eind druk	-400.00 daPa
Druk	-84.00 daPa	Pomp snelheid	Maximaal
Gradiënt	NaN ml	Test toon	226 Hz

ECV.	0.00 ml	Begin druk	0.00 daPa
Compliantie	0.00 ml	Eind druk	0.00 daPa
Druk	0.00 daPa	Pomp snelheid	N/A
Gradiënt	0.00 ml	Test toon	N/A

Patient ID: 10

Sex: male

Age: 19 months

Supplement 1. Audiological assessments per patient

DP-OAE Test Report – PID 10

Right ear

Ear canal frequency response

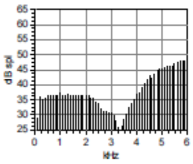

DPOAE response

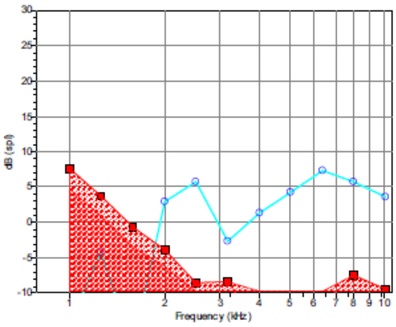

Test Summary
Sum all 1/2 octave = 11,8dBspl
Ave DP 1/2oct (1-6) = 2,7dBspl

Half octave band OAE power

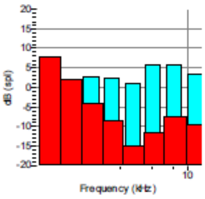

Freq (kHz)	Signal (dBspl)	Noise (dBspl)	SNR (dB)
1.0	-14,1	7,6	-21,7
1.4	-8,5	1,8	-10,4
2.0	3,0	-3,9	6,9
2.8	2,5	-8,5	11,0
4.0	1,3	-15,0	16,3
6.0	5,9	-11,6	17,5
8.0	5,7	-7,4	13,1
10.0	3,6	-9,5	13,1

Test Environment
NLo = 487 NHi = 41 RejLev = 8mPa, 52,0dBspl Test time = 51s
Hardware= USBOAE Probe = Probe 1

Left ear

Ear canal frequency response

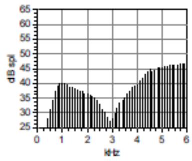

DPOAE response

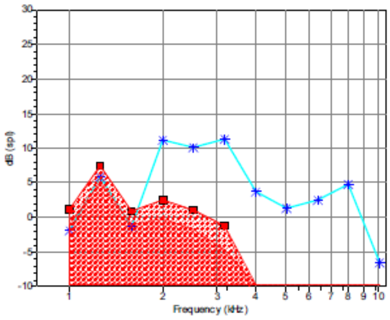

Test Summary
Sum all 1/2 octave = 15,1dBspl
Ave DP 1/2oct (1-6) = 6,1dBspl

Half octave band OAE power

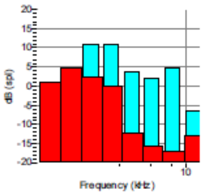

Freq (kHz)	Signal (dBspl)	Noise (dBspl)	SNR (dB)
1.0	-1,8	1,2	-3,0
1.4	2,9	4,8	-1,9
2.0	11,2	2,5	8,7
2.8	10,8	0,0	10,7
4.0	3,8	-12,2	16,0
6.0	1,9	-15,6	17,5
8.0	4,8	-16,8	21,6
10.0	-6,5	-13,1	6,6

Test Environment
NLo = 712 NHi = 232 RejLev = 8mPa, 52,0dBspl Test time = 91s
Hardware= USBOAE Probe = Probe 1

Right

Freq Hz	L1 dBspl	L2 dBspl	DP dBspl	2SD Noise dBspl	1SD Noise dBspl	SNR dB
1001	67,3	57,1	-14,1	7,6	4,7	-21,7
1257	65,4	56,9	-4,2	3,8	1,0	-8,0
1587	65,7	56,4	-17,6	-0,7	-3,4	-16,9
2002	65,5	56,1	3,0	-3,9	-6,3	6,9
2515	65,8	51,1	5,7	-8,6	-11,2	14,3
3174	65,0 *	55,0 *	-2,6	-8,4	-11,1	5,8
4004	65,0 *	55,8	1,3	-15,0	-17,4	16,3
5042	66,7	57,0	4,2	-11,6	-13,4	15,8
6348	67,0	57,3	7,3	-11,6	-14,3	18,9
7996	67,3	55,0 *	5,7	-7,4	-10,0	13,1
10083	67,2	55,0 *	3,6	-9,5	-11,9	13,1

Left

Freq Hz	L1 dBspl	L2 dBspl	DP dBspl	2SD Noise dBspl	1SD Noise dBspl	SNR dB
1001	65,0 *	52,6	-1,8	1,2	-1,1	-3,0
1257	63,4	57,3	5,8	7,5	5,2	-1,7
1587	65,9	55,0 *	-1,4	0,9	-1,1	-2,3
2002	65,0 *	55,0 *	11,2	2,5	-0,1	8,7
2515	66,4	52,5	10,2	1,1	-1,7	9,1
3174	65,0 *	55,0 *	11,3	-1,2	-4,6	12,5
4004	65,0 *	55,8	3,8	-12,2	-15,1	16,0
5042	65,4	56,4	1,3	-16,1	-18,4	17,4
6348	66,7	56,7	2,5	-15,1	-16,9	17,6
7996	66,1	57,2	4,8	-16,8	-18,4	21,6
10083	66,0	55,0 *	-6,5	-13,1	-15,3	6,6

TEOAE Test Report – PID 10

Right

Test type: TE - QuickScreen
Stimulus: 83,4dB peSPL
Mode: Gen Diag
Tester ID: AUD
Data file: 1ROV3O32.DTA
Notes:

Response waveform

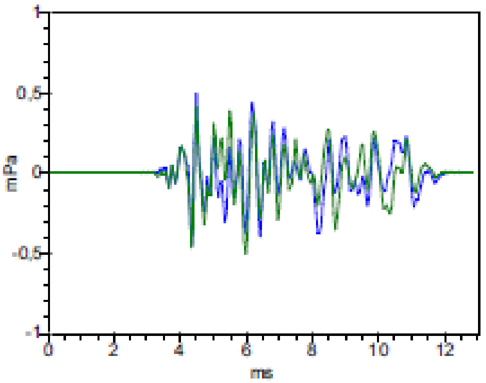

Half octave band OAE power

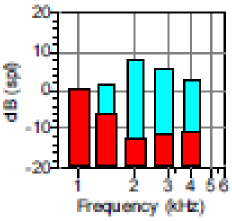

Freq (kHz)	Signal (dB spl)	Noise (dBspl)	SNR (dB)
1.0	-3,9	0,4	-4,3
1.4	1,2	-5,8	7,0
2.0	7,8	-12,1	19,9
2.8	5,3	-11,1	16,4
4.0	2,5	-10,7	13,2

Test Summary
Total OAE response = 11,0dBspl Total Noise = 6,7dBspl

Checkfit stimulus

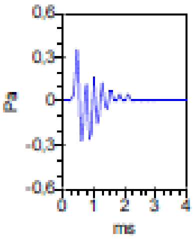

Ear canal response

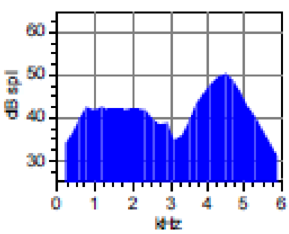

Test Environment
NLo = 138 NHi = 4 Test time = 37s
RejLev = 52,0dBspl Repro = 73% Stim stab = 98%
Hardware USBOAE Probe = Probe 1

Left

Test type: TE - QuickScreen
Stimulus: 85,3dB peSPL
Mode: Gen Diag
Tester ID: AUD
Data file: 1ROV3O34.DTA
Notes:

Response waveform

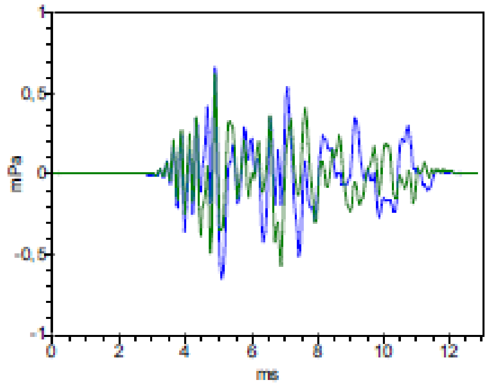

Half octave band OAE power

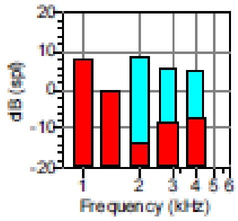

Freq (kHz)	Signal (dB spl)	Noise (dBspl)	SNR (dB)
1.0	-9,7	7,6	-17,3
1.4	-1,2	0,1	-1,3
2.0	8,5	-13,4	22,0
2.8	5,2	-8,7	13,9
4.0	5,0	-7,2	12,2

Test Summary
Total OAE response = 11,4dBspl Total Noise = 11,5dBspl

Checkfit stimulus

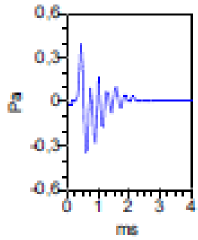

Ear canal response

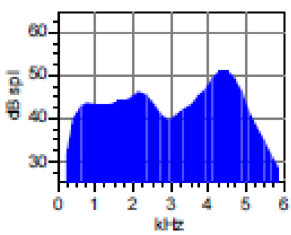

Test Environment
NLo = 260 NHi = 89 Test time = 79s
RejLev = 52,0dBspl Repro = 46% Stim stab = 95%
Hardware = USBOAE Probe = Probe 1

Tympanometry – PID 10

Tympanogram (Tymp)

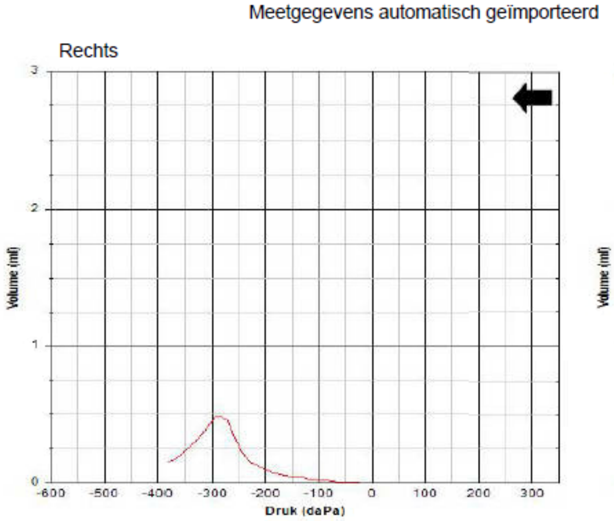

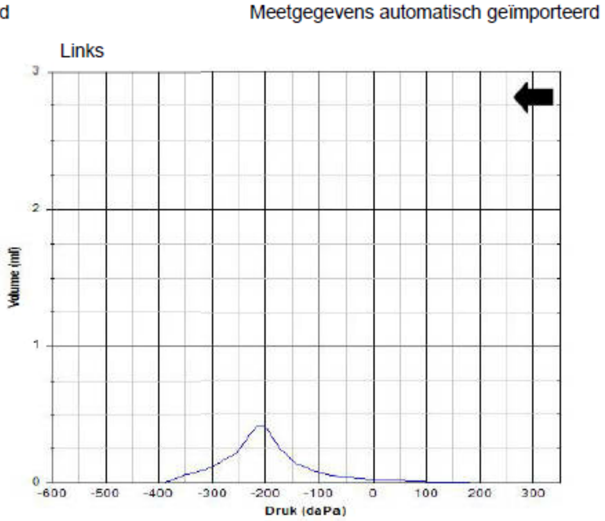

ECV.	0.62 ml	Begin druk	200.00 daPa
Compliantie	32.70 ml	Eind druk	-400.00 daPa
Druk	0.00 daPa	Pomp snelheid	Maximaal
Gradiënt	NaN ml	Test toon	226 Hz

ECV.	0.60 ml	Begin druk	200.00 daPa
Compliantie	0.42 ml	Eind druk	-400.00 daPa
Druk	-205.00 daPa	Pomp snelheid	Maximaal
Gradiënt	NaN ml	Test toon	226 Hz

Patient ID: 11

Sex: male

Age: 24 months

DP-OAE Test Report – PID 11

Right ear

Left ear

DPOAE's left and right not performed

TEOAE Test Report – PID 11

Right

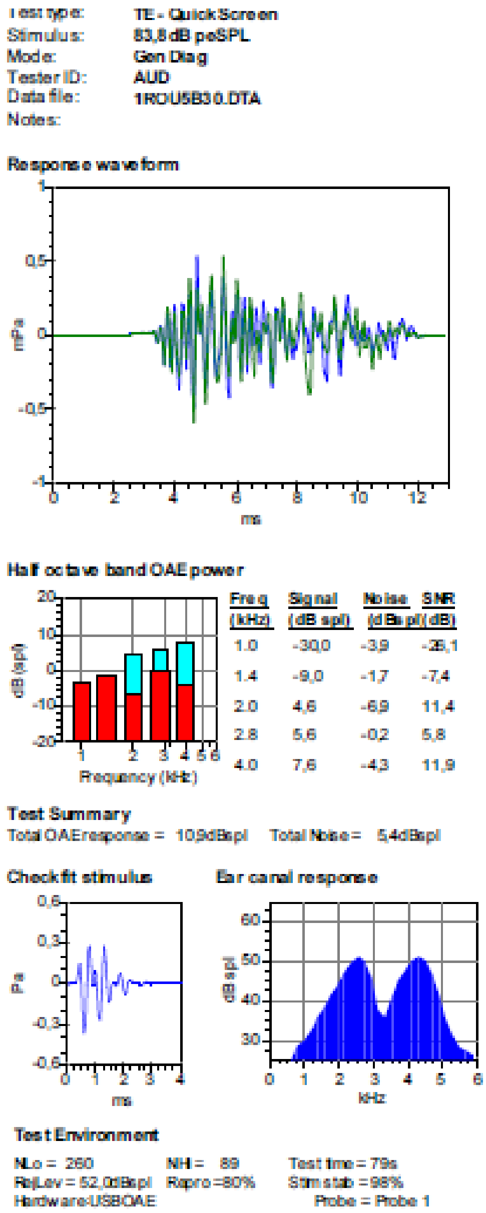

Test type: TE - QuickScreen
Stimulus: 83,8dB peSPL
Mode: Gen Diag
Tester ID: AUD
Data file: 1ROU5B30.DTA
Notes:

Response waveform

Half octave band OAE power

Freq (kHz)	Signal (dB spl)	Noise (dB spl)	SNR (dB)
1.0	-30,0	-3,9	-26,1
1.4	-9,0	-1,7	-7,4
2.0	4,6	-6,9	11,4
2.8	5,6	-0,2	5,8
4.0	7,6	-4,3	11,9

Test Summary
Total OAE response = 10,9dBspl Total Noise = 5,4dBspl

Checkfit stimulus Ear canal response

Test Environment
NLo = 260 NH = 89 Test time = 79s
RejLev = 52,0dBspl Repro = 80% Stim stab = 98%
Hardware=USBOAE Probe = Probe 1

Left

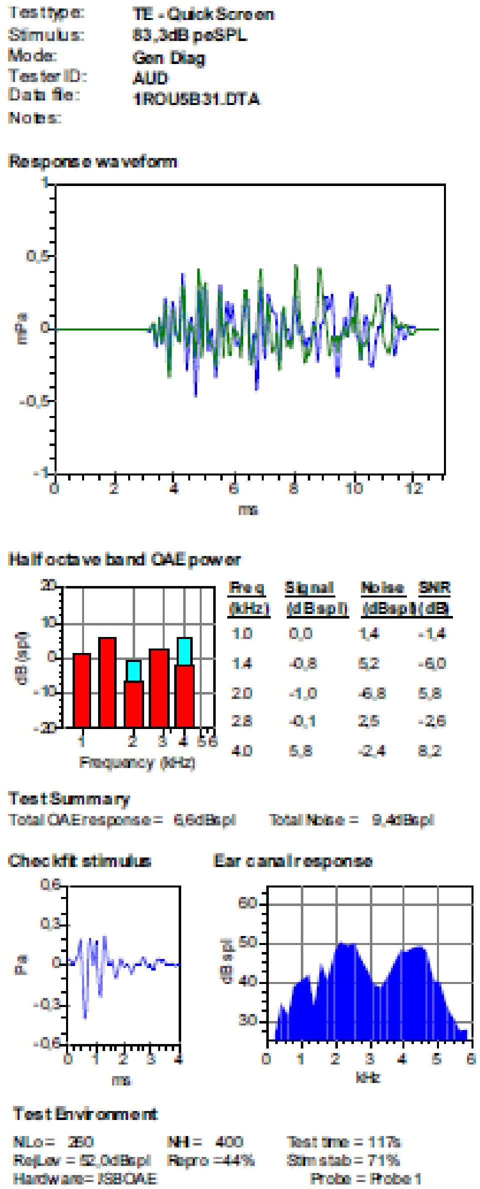

Test type: TE - QuickScreen
Stimulus: 83,3dB peSPL
Mode: Gen Diag
Tester ID: AUD
Data file: 1ROU5B31.DTA
Notes:

Response waveform

Half octave band OAE power

Freq (kHz)	Signal (dB spl)	Noise (dB spl)	SNR (dB)
1.0	0,0	1,4	-1,4
1.4	-0,8	5,2	-6,0
2.0	-1,0	-6,8	5,8
2.8	-0,1	2,5	-2,6
4.0	5,8	-2,4	8,2

Test Summary
Total OAE response = 6,6dBspl Total Noise = 9,4dBspl

Checkfit stimulus Ear canal response

Test Environment
NLo = 260 NH = 400 Test time = 117s
RejLev = 52,0dBspl Repro = 44% Stim stab = 71%
Hardware= USBOAE Probe = Probe 1

Supplement 1. Audiological assessments per patient

Tympanometry – PID 11

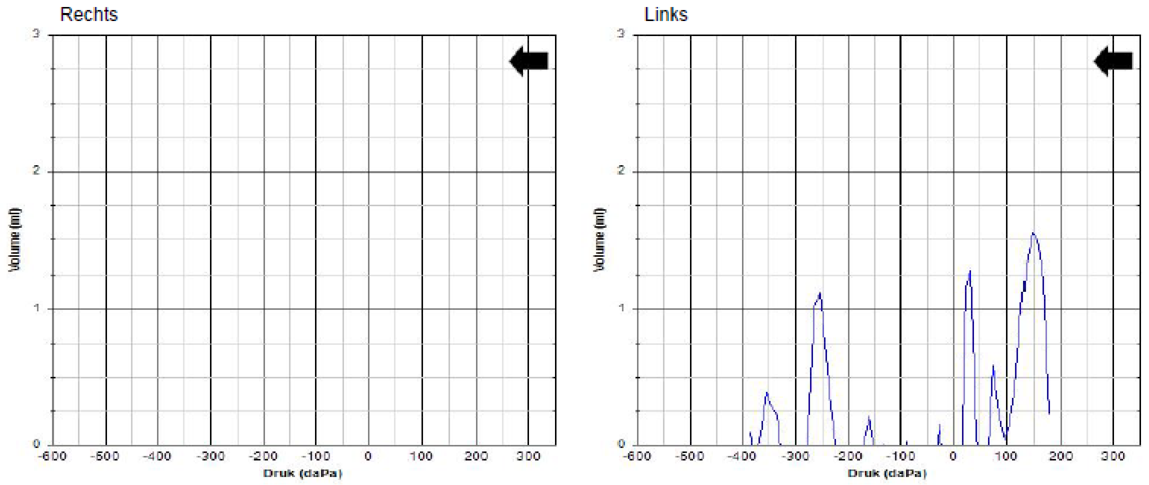

ECV.	3.45 ml	Begin druk	200.00 daPa	ECV.	4.56 ml	Begin druk	200.00 daPa
Compliantie	0.00 ml	Eind druk	-400.00 daPa	Compliantie	1.55 ml	Eind druk	-400.00 daPa
Druk	0.00 daPa	Pomp snelheid	Maximaal	Druk	147.00 daPa	Pomp snelheid	Maximaal
Gradiënt	NaN ml	Test toon	226 Hz	Gradiënt	NaN ml	Test toon	226 Hz
