# Supplementary material for: Exploring and Overcoming Challenges for Efficient Audiological Testing in Children Under 5 Years of Age—Screening with Otoacoustic Emissions
Source: Audiol Res. 2026 May 15;16(3):74. doi: 10.3390/audiolres16030074 (PMC13214472; doi:10.3390/audiolres16030074)
Supplement: Supplementary file 1 [file audiolres-16-00074-s001.zip › File_S2.pdf]

Supplement 2. Evaluation sheet sent out to audiological experts

Evaluation sheet – PID 01

To fill in by audiologist or audiological physician based on his/her expertise.

DPOAE - **Fill the applicable box for the right and left ear with an 'X'.**

DPOAE (Based on SNR)	Right ear	Left ear
Present (< 4 kHz)		
Absent (< 4 kHz)		
Cannot be interpreted* (< 4 kHz)		
Present (4 – 6 kHz)		
Absent (4 – 6 kHz)		
Cannot be interpreted* (4 - 6 kHz)		
Present (> 6 kHz)		
Absent (> 6 kHz)		
Cannot be interpreted* (> 6 kHz)		
Emissions present from XX to XX kHz		

TEOAE – Fill the applicable box for the right and left ear with an 'X'.

TEOAE (Based on SNR)	Right ear	Left ear
Present		
Absent		
Cannot be interpreted*		

***In case of results that cannot be interpreted, reason ....**
...

Based on the OAE's, would you have performed a tympanometry in your department? **Fill the applicable box for the right and left ear with an 'X'.**

Tympanometry necessary?	Right ear	Left ear
Yes^		
No		

^ Only if OAE's were absent in abovementioned tables.

Supplement 2. Evaluation sheet sent out to audiological experts

Tympanometry
Fill the applicable box for the right and left ear with an 'X'.

Tympanometry interpretation	Right ear	Left ear
Normal**		
Abnormal		
Cannot be performed, because child was not cooperative		

****Cut-off middle ear pressure -200 daPa (by consensus meeting December 3, 2021).**

Optional: Tympanometry classification

☐ Type A

☐ Type As

☐ Type Ad

☐ Type B

☐ Type C

Overall conclusion
Fill the applicable box for the right and left ear with an 'X'.

Suspicious of hearing loss		Right ear	Left ear
Yes			
	sensorineural		
	conductive		
	mixed		
No			
Unclear			

Would you have performed more audiological tests to rule out ototoxicity, if this child was sent to your audiological department? If yes: check which test(s) you would have performed.

☐ **No**

☐ Yes, I would have performed:

- ☐ Automatic Auditory Brainstem Response (AABR)
- ☐ Visual Reinforcement Audiometry (VRA)
- ☐ Conditioned Play Audiometry (CPA)
- ☐ Pure Tone Audiogram (PTA)
- ☐ Speech Audiometry

☐ Other: ....
