# Supplementary material for: Exploring and Overcoming Challenges for Efficient Audiological Testing in Children Under 5 Years of Age—Screening with Otoacoustic Emissions
Source: Audiol Res. 2026 May 15;16(3):74. doi: 10.3390/audiolres16030074 (PMC13214472; doi:10.3390/audiolres16030074)

Patient ID: 01

No follow-up testing performed

Patient ID: 02

Sex: female

Age at follow-up: 36 months

Follow-up Tympanometry – PID 02

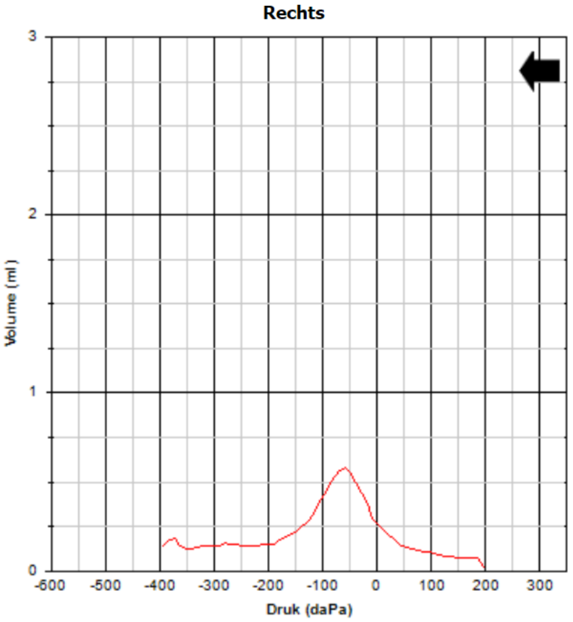

**ECV.**	0.42 ml	**Begin druk**	200.00 daPa
**Compliantie**	0.58 ml	**Eind druk**	-400.00 daPa
**Druk**	-57.00 daPa	**Pomp snelheid**	Maximaal
**Gradiënt**	199.00 ml	**Test toon**	226 Hz

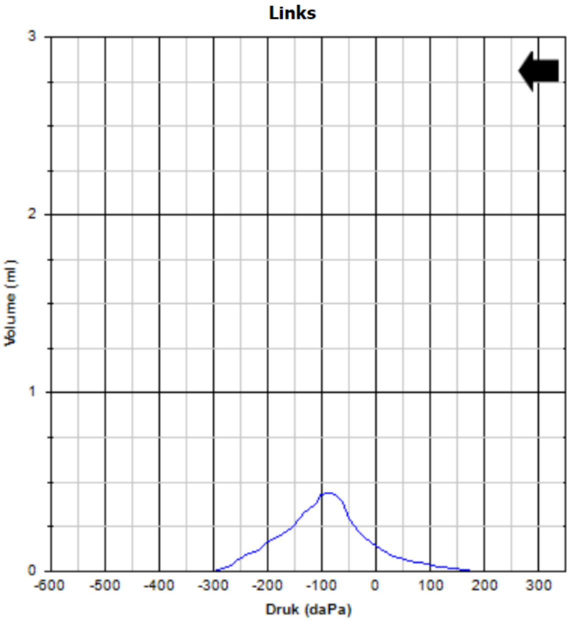

**ECV.**	0.42 ml	**Begin druk**	200.00 daPa
**Compliantie**	0.44 ml	**Eind druk**	-400.00 daPa
**Druk**	-90.00 daPa	**Pomp snelheid**	Maximaal
**Gradiënt**	144.00 ml	**Test toon**	226 Hz

Supplement 3. Follow-up audiological assessments per patient

Follow-up PTA test report – PID 02

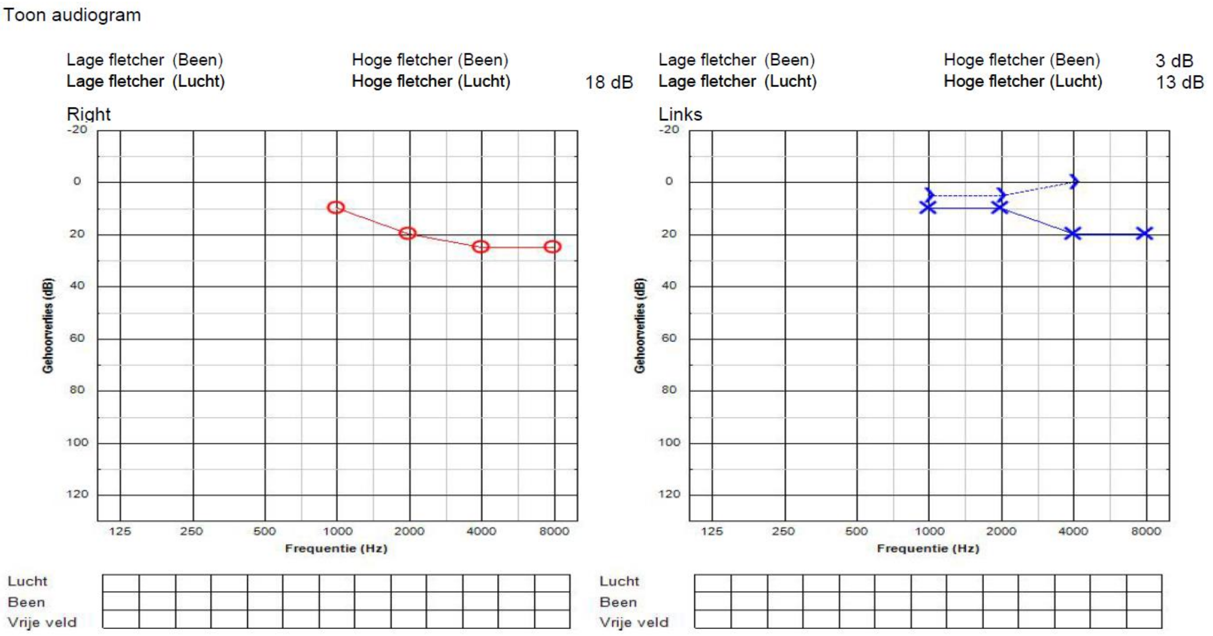

Patient ID: 03

Sex: female

Age at follow-up: 6 years and 3 months

Follow-up Tympanometry – PID 03

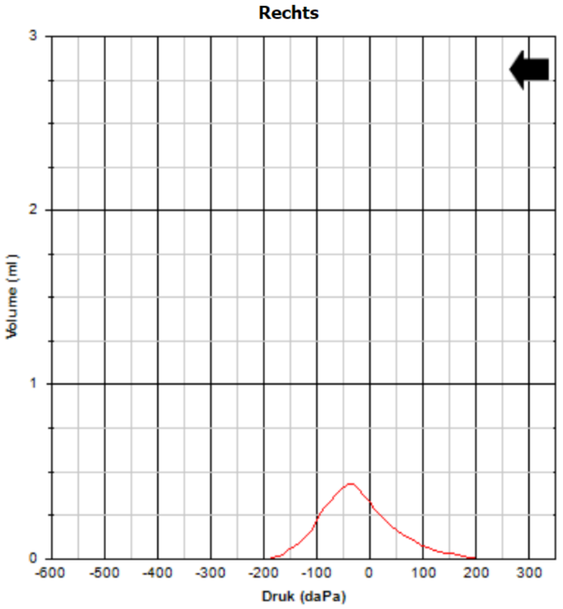

**ECV.**	0.78 ml	**Begin druk**	200.00 daPa
**Compliantie**	0.42 ml	**Eind druk**	-400.00 daPa
**Druk**	-30.00 daPa	**Pomp snelheid**	Maximaal
**Gradiënt**	139.00 ml	**Test toon**	226 Hz

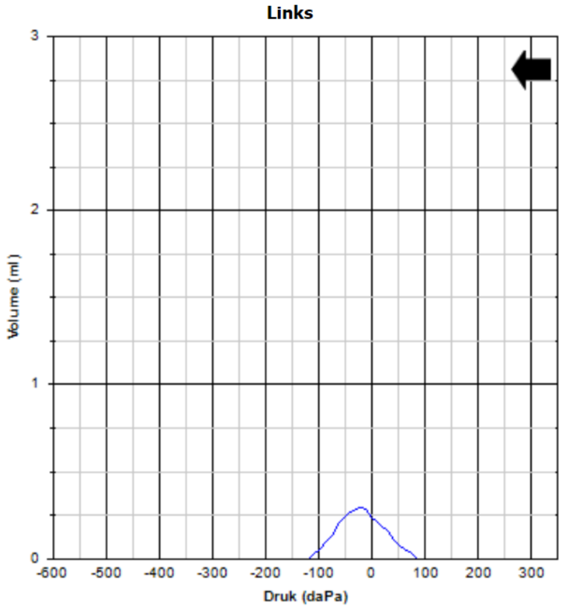

**ECV.**	0.97 ml	**Begin druk**	200.00 daPa
**Compliantie**	0.29 ml	**Eind druk**	-400.00 daPa
**Druk**	-20.00 daPa	**Pomp snelheid**	Maximaal
**Gradiënt**	132.00 ml	**Test toon**	226 Hz

Supplement 3. Follow-up audiological assessments per patient

Follow-up PTA test report – PID 03

Toon audiogram

Lage fletcher (Lucht) 3 dB **Hoge fletcher (Lucht)** 7 dB

Lage fletcher (Lucht) 0 dB **Hoge fletcher (Lucht)** 0 dB

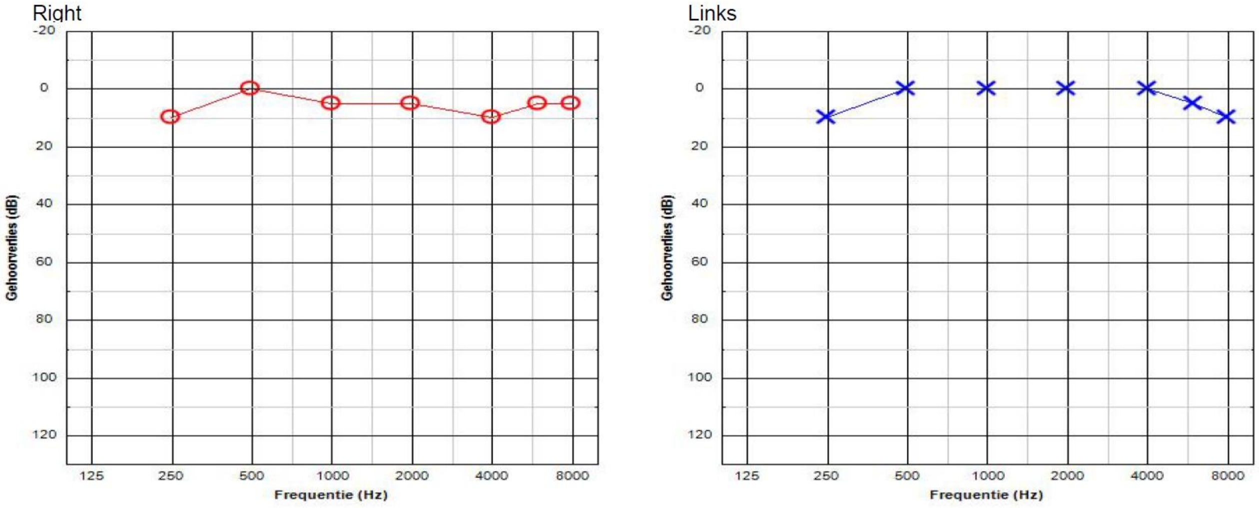

Lucht													
Been													
Vrije veld													

Lucht													
Been													
Vrije veld													

Patient ID: 04

Sex: male

Age at follow-up: 5 years 11 months

Follow-up Tympanometry – PID 04

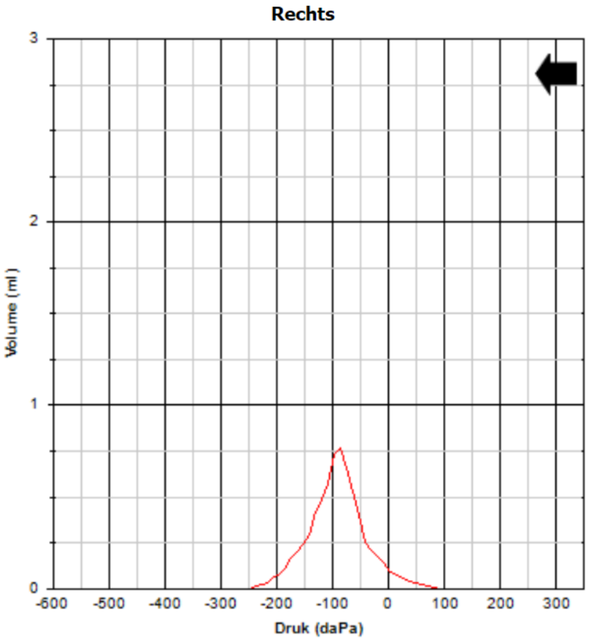

**ECV.**	0.74 ml	**Begin druk**	200.00 daPa
**Compliantie**	0.76 ml	**Eind druk**	-400.00 daPa
**Druk**	-84.00 daPa	**Pomp snelheid**	Maximaal
**Gradiënt**	493.00 ml	**Test toon**	226 Hz

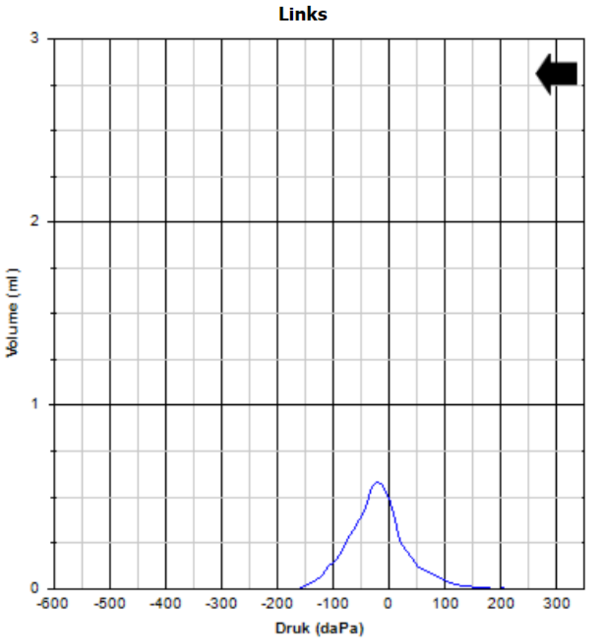

**ECV.**	0.61 ml	**Begin druk**	200.00 daPa
**Compliantie**	0.58 ml	**Eind druk**	-400.00 daPa
**Druk**	-20.00 daPa	**Pomp snelheid**	Maximaal
**Gradiënt**	317.00 ml	**Test toon**	226 Hz

Supplement 3. Follow-up audiological assessments per patient

Follow-up PTA test report – PID 04

Toon audiogram

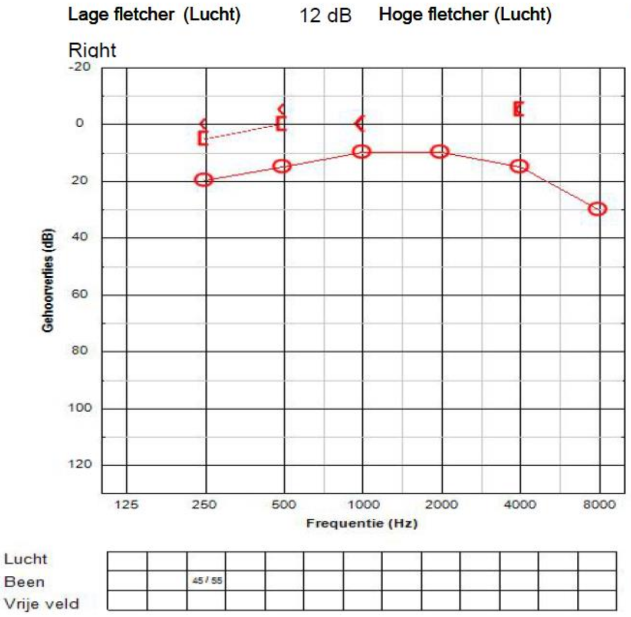

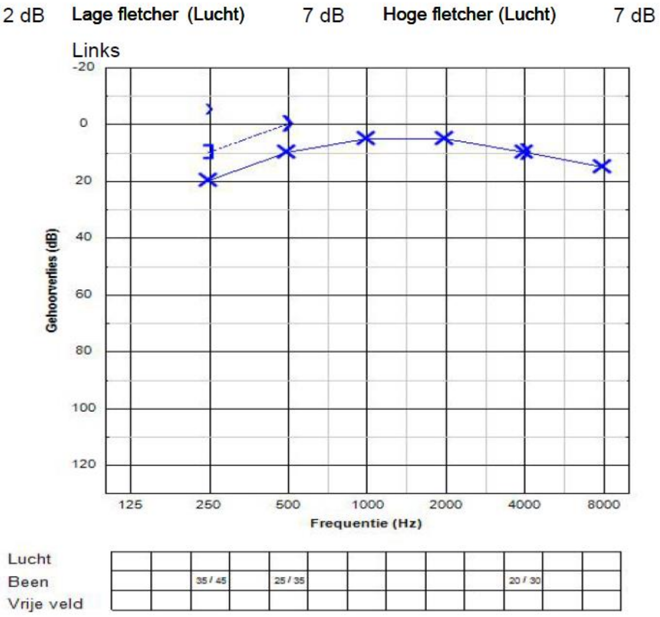

Hoog frequentie audiogram

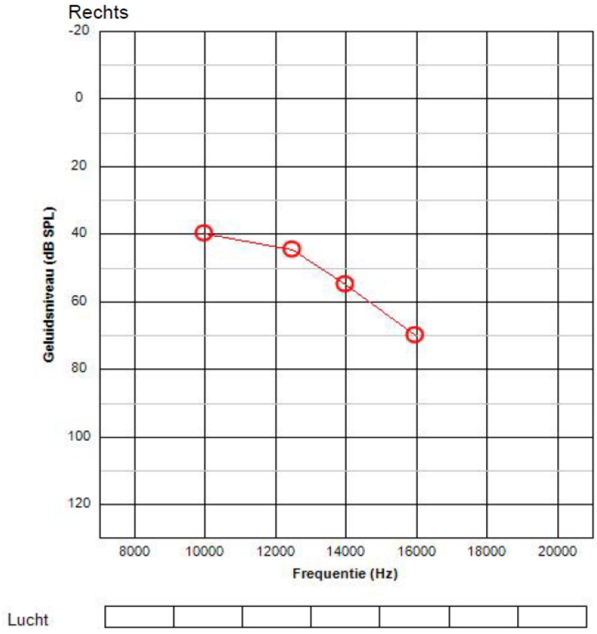

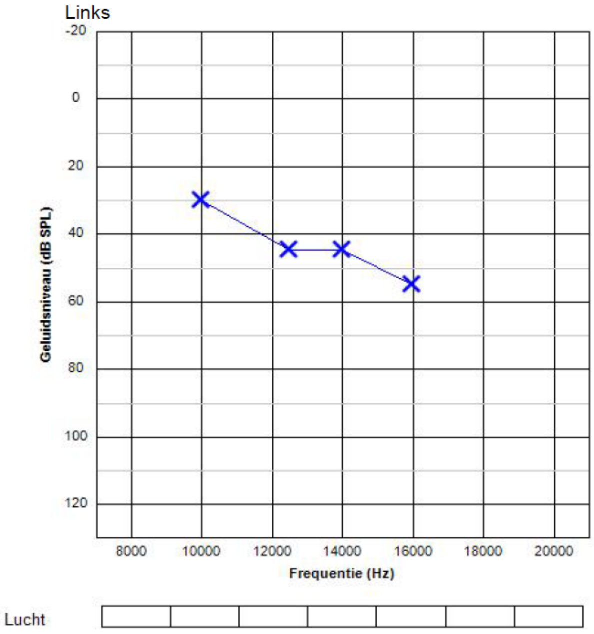

Patient ID: 05

No follow-up testing performed

Patient ID: 06

Sex: female

Age at follow-up: 3 years and 2 months

Follow-up Tympanometry – PID 06

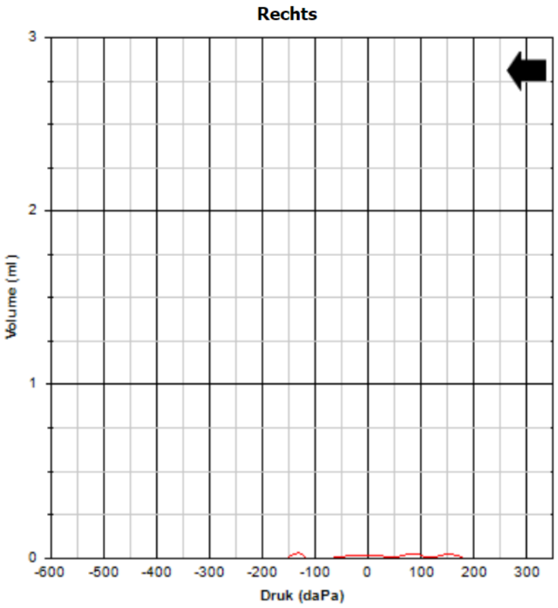

**ECV.**	0.45 ml	**Begin druk**	200.00 daPa
**Compliantie**	0.03 ml	**Eind druk**	-400.00 daPa
**Druk**	-130.00 daPa	**Pomp snelheid**	Maximaal
**Gradiënt**	327.00 ml	**Test toon**	226 Hz

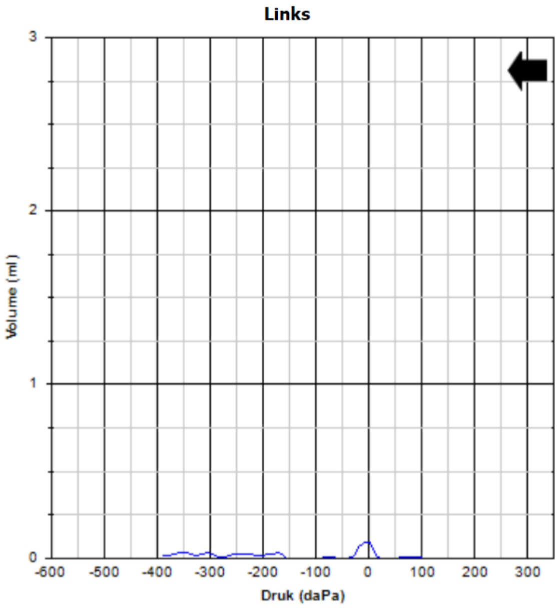

**ECV.**	0.29 ml	**Begin druk**	200.00 daPa
**Compliantie**	0.09 ml	**Eind druk**	-400.00 daPa
**Druk**	4.00 daPa	**Pomp snelheid**	Maximaal
**Gradiënt**	327.00 ml	**Test toon**	226 Hz

Supplement 3. Follow-up audiological assessments per patient

Follow-up VRA test report – PID 06

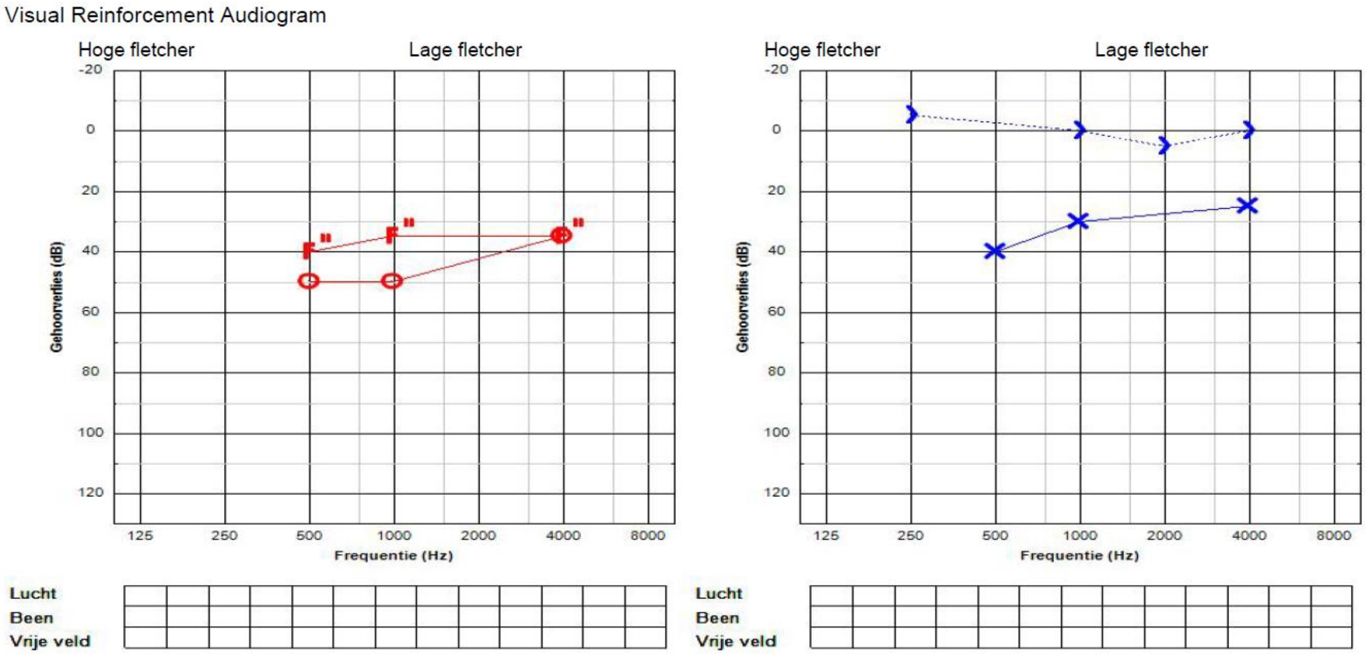

Patient ID: 07

Sex: female

Age at follow-up: 3 years 2 months

Follow-up Tympanometry – PID 07

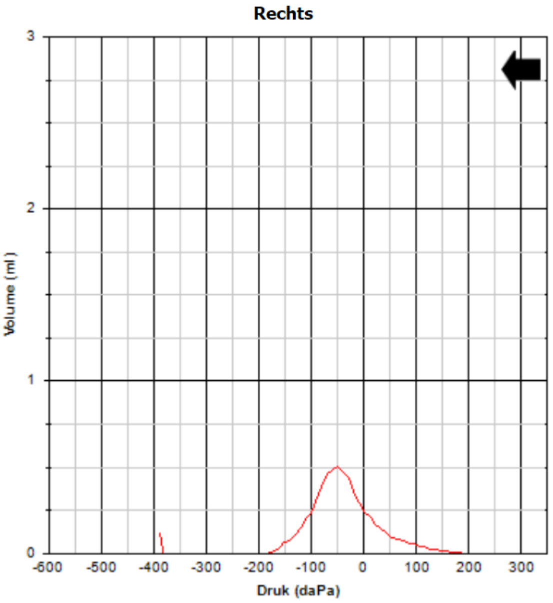

**ECV.**	0.64 ml	**Begin druk**	200.00 daPa
**Compliantie**	0.50 ml	**Eind druk**	-400.00 daPa
**Druk**	-48.00 daPa	**Pomp snelheid**	Maximaal
**Gradiënt**	241.00 ml	**Test toon**	226 Hz

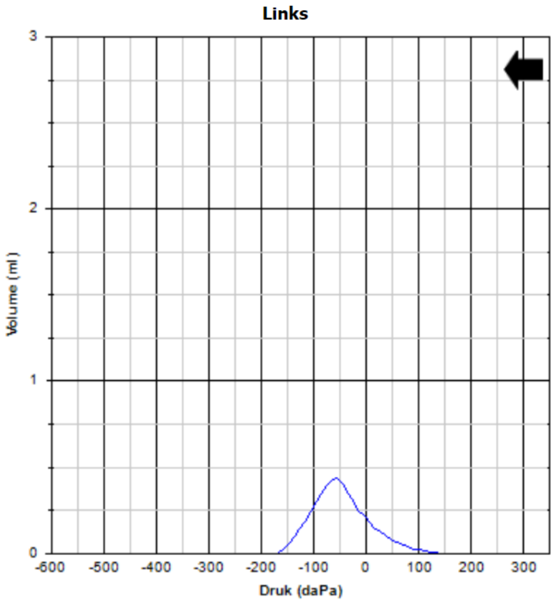

**ECV.**	0.72 ml	**Begin druk**	200.00 daPa
**Compliantie**	32.70 ml	**Eind druk**	-400.00 daPa
**Druk**	0.00 daPa	**Pomp snelheid**	Maximaal
**Gradiënt**	327.00 ml	**Test toon**	226 Hz

Supplement 3. Follow-up audiological assessments per patient

Follow-up VRA test report – PID 07

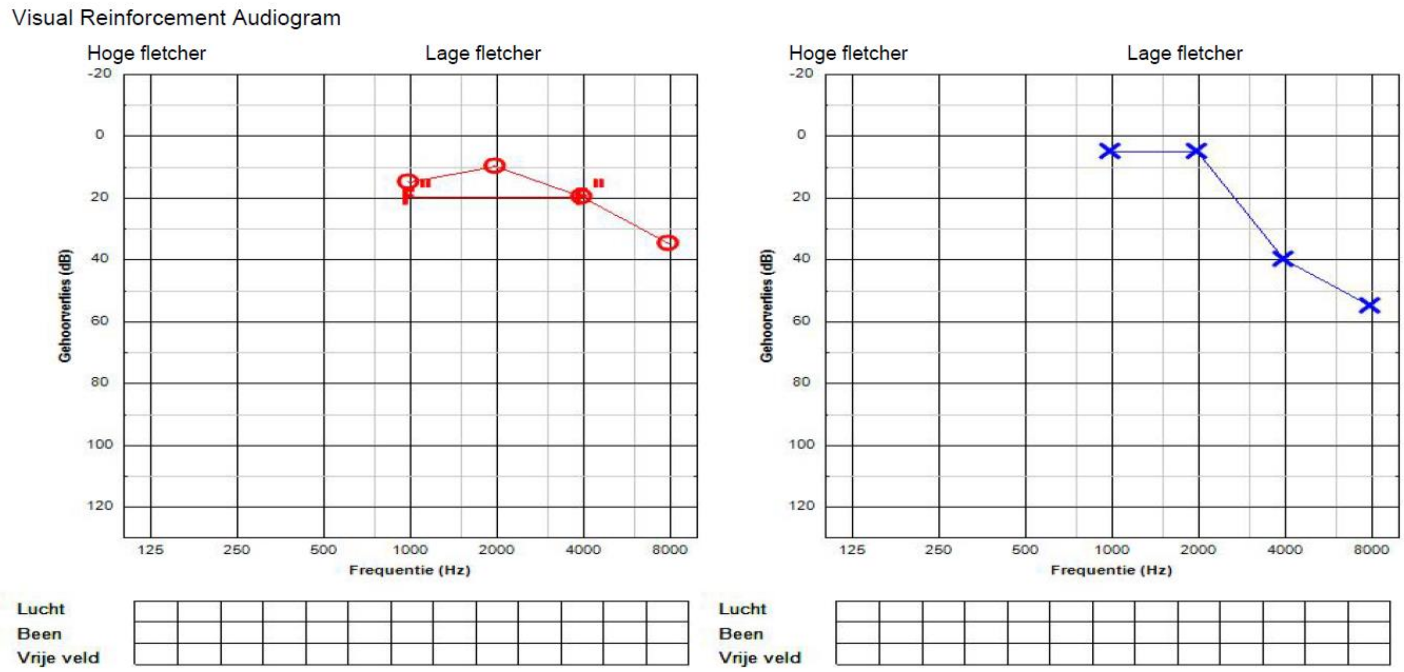

Patient ID: 08

Sex: female

Age at follow-up: 3 years 3 months

Follow-up Tympanometry – PID 08

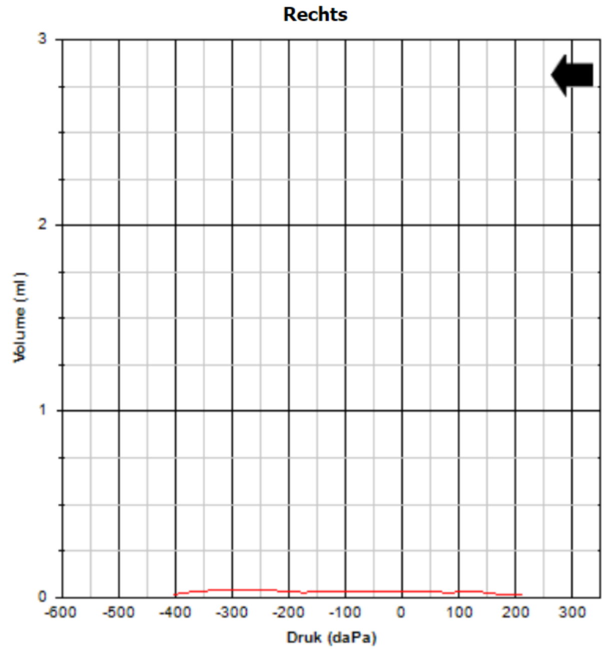

**ECV.**	0.54 ml	**Begin druk**	200.00 daPa
**Compliantie**	0.04 ml	**Eind druk**	-400.00 daPa
**Druk**	-296.00 daPa	**Pomp snelheid**	Maximaal
**Gradiënt**	6.00 ml	**Test toon**	226 Hz

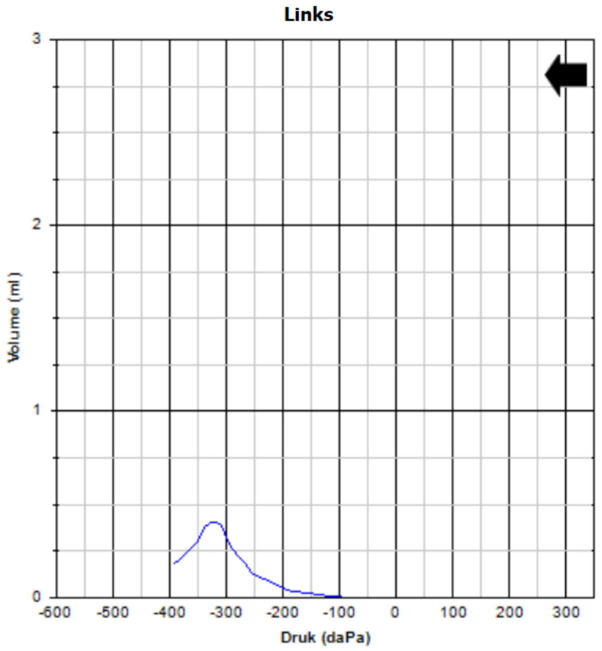

**ECV.**	0.52 ml	**Begin druk**	200.00 daPa
**Compliantie**	0.40 ml	**Eind druk**	-400.00 daPa
**Druk**	-317.00 daPa	**Pomp snelheid**	Maximaal
**Gradiënt**	18.00 ml	**Test toon**	226 Hz

Supplement 3. Follow-up audiological assessments per patient

Follow-up VRA test report – PID 08

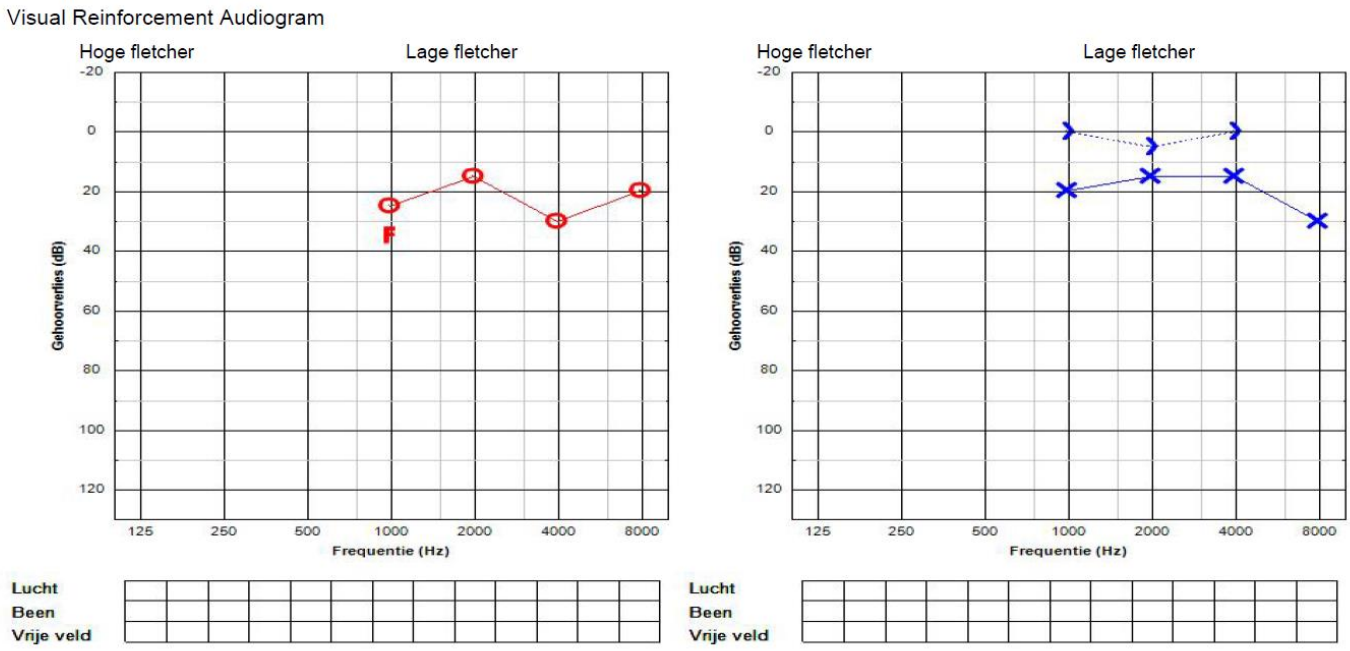

Patient ID: 09

Sex: male

Age at follow-up: 3 years 3 months

Follow-up Tympanometry – PID 09

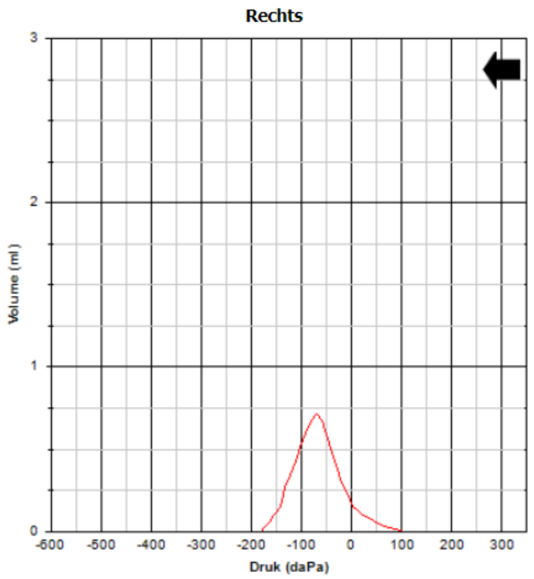

**ECV.**	0.60 ml	**Begin druk**	200.00 daPa
**Compliantie**	0.71 ml	**Eind druk**	-400.00 daPa
**Druk**	-69.00 daPa	**Pomp snelheid**	Maximaal
**Gradiënt**	354.00 ml	**Test toon**	226 Hz

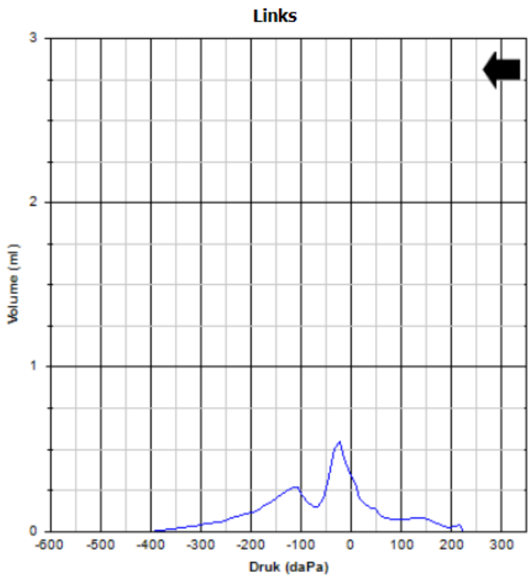

**ECV.**	0.38 ml	**Begin druk**	200.00 daPa
**Compliantie**	0.54 ml	**Eind druk**	-400.00 daPa
**Druk**	-22.00 daPa	**Pomp snelheid**	Maximaal
**Gradiënt**	369.00 ml	**Test toon**	226 Hz

Supplement 3. Follow-up audiological assessments per patient

Follow-up VRA test report – PID 09

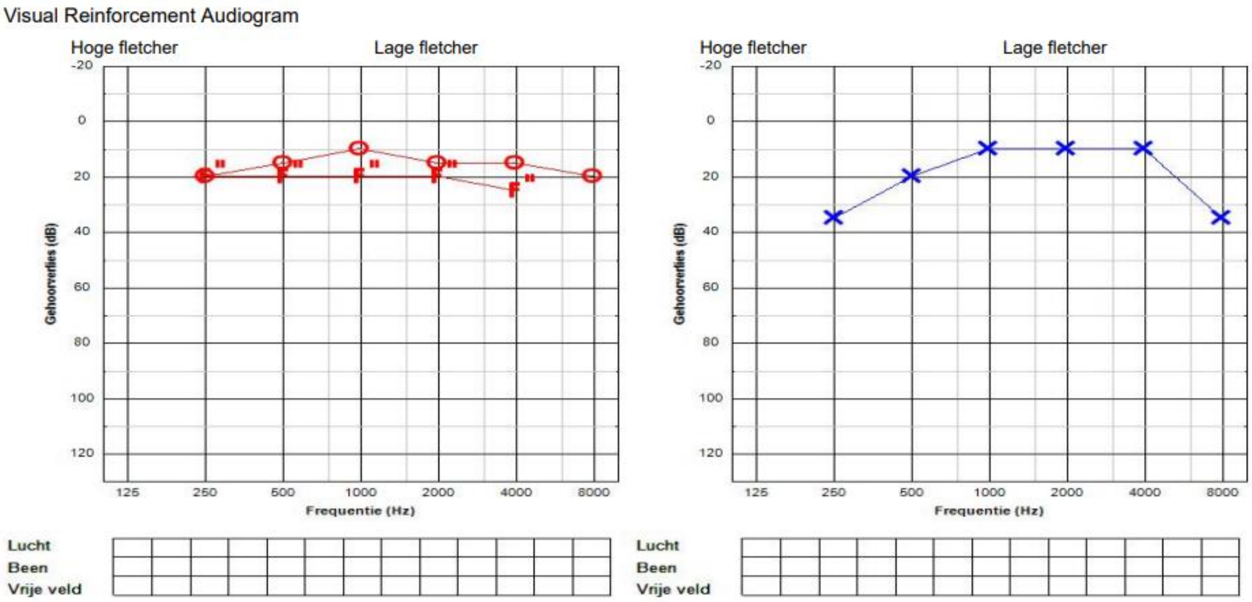

Patient ID: 10

Sex: male

Age at follow-up: 3 years 3 months

Follow-up Tympanometry – PID 10

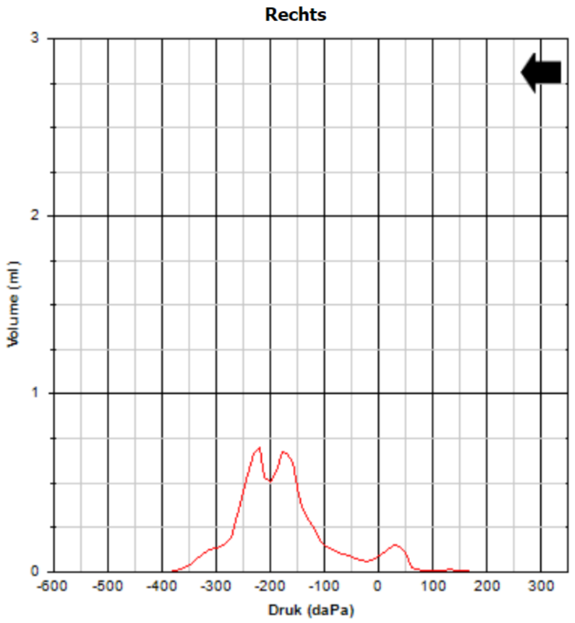

**ECV.**	0.64 ml	**Begin druk**	200.00 daPa
**Compliantie**	0.70 ml	**Eind druk**	-400.00 daPa
**Druk**	-219.00 daPa	**Pomp snelheid**	Maximaal
**Gradiënt**	268.00 ml	**Test toon**	226 Hz

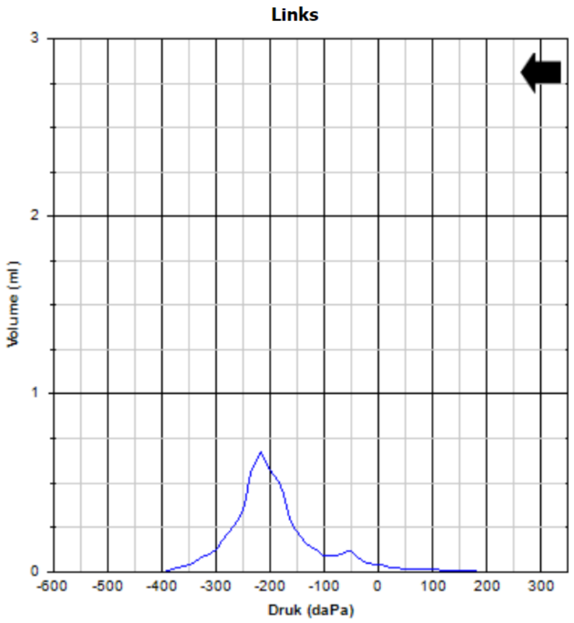

**ECV.**	0.59 ml	**Begin druk**	200.00 daPa
**Compliantie**	0.67 ml	**Eind druk**	-400.00 daPa
**Druk**	-214.00 daPa	**Pomp snelheid**	Maximaal
**Gradiënt**	323.00 ml	**Test toon**	226 Hz

Supplement 3. Follow-up audiological assessments per patient

Follow-up VRA test report – PID 10

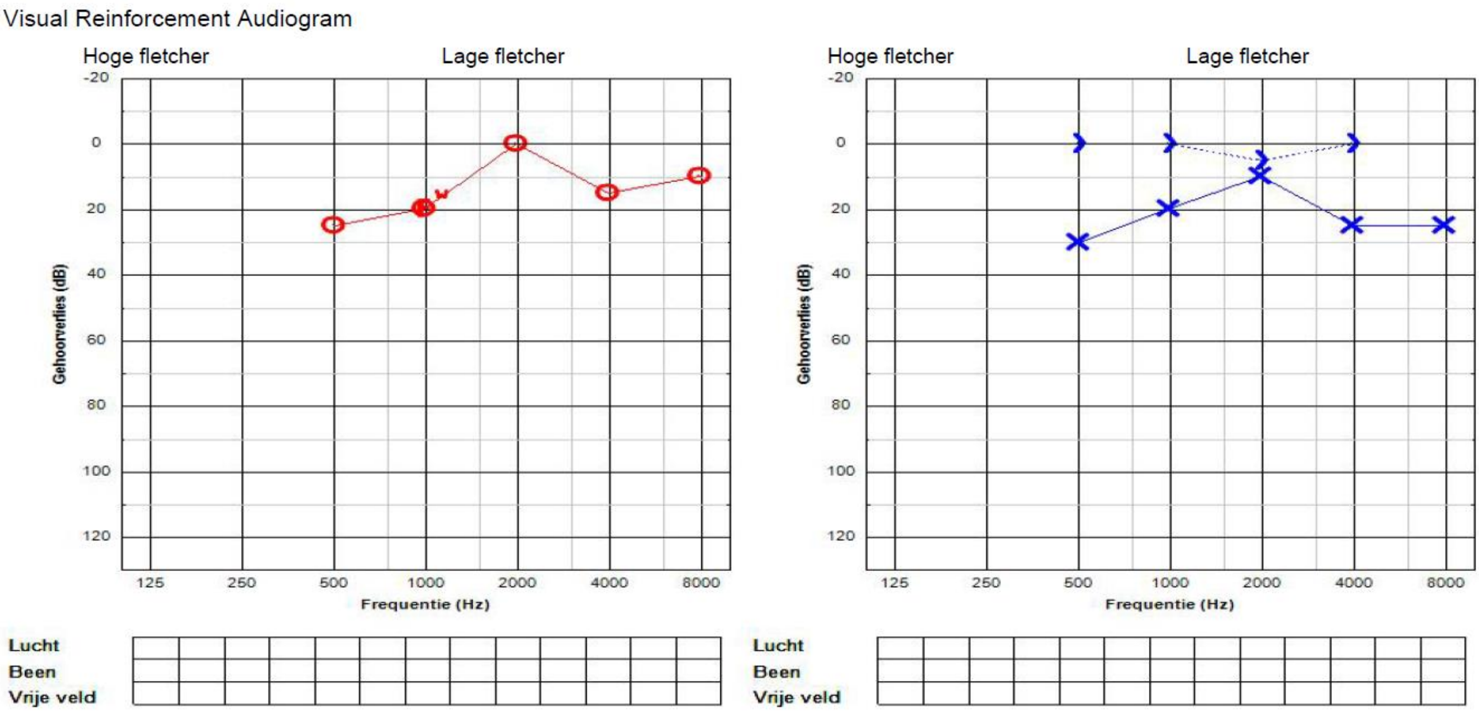

Patient ID: 11

Sex: male

Age: 3 years

Follow-up Tympanometry – PID 11

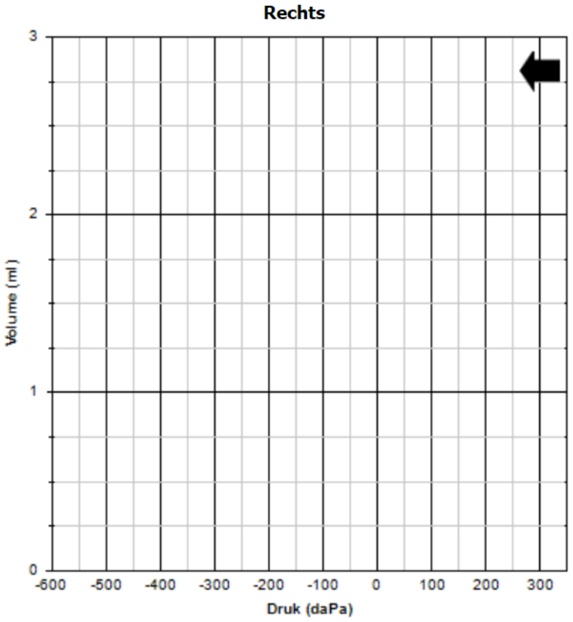

**ECV.**	0.49 ml	**Begin druk**	200.00 daPa
**Compliantie**	32.70 ml	**Eind druk**	-400.00 daPa
**Druk**	0.00 daPa	**Pomp snelheid**	Maximaal
**Gradiënt**	327.00 ml	**Test toon**	226 Hz

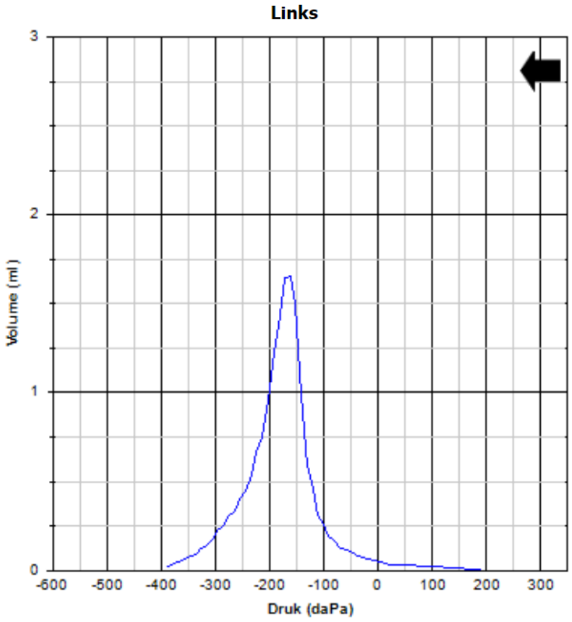

**ECV.**	0.54 ml	**Begin druk**	200.00 daPa
**Compliantie**	1.65 ml	**Eind druk**	-400.00 daPa
**Druk**	-160.00 daPa	**Pomp snelheid**	Maximaal
**Gradiënt**	105.00 ml	**Test toon**	226 Hz

Supplement 3. Follow-up audiological assessments per patient

Follow-up VRA test report – PID 11

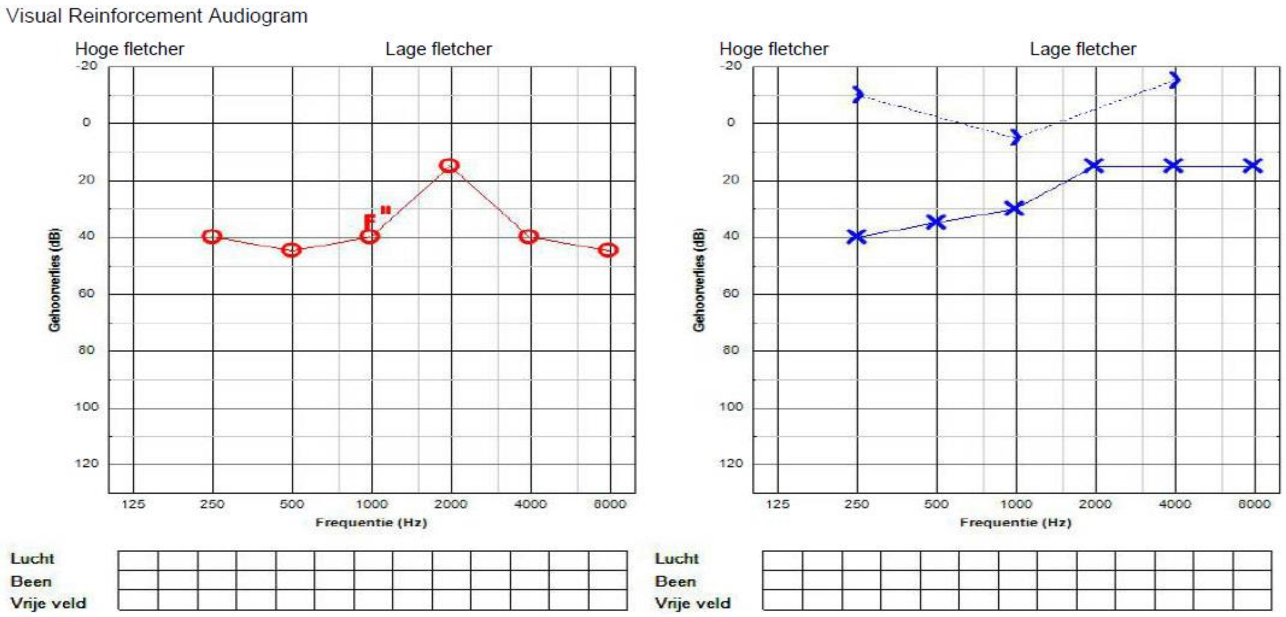

Supplement: Supplementary file 1 [file audiolres-16-00074-s001.zip › File_S3.pdf]
